# Evaluation of Organic and Inorganic Foulant Interaction Using Modified Fouling Models in Constant Flux Dead-End Operation with Microfiltration Membranes

**DOI:** 10.3390/membranes13110853

**Published:** 2023-10-25

**Authors:** Muhammad Qasim, Ali Akbar, Imtiaz Afzal Khan, Mumtaz Ali, Eui-Jong Lee, Kang Hoon Lee

**Affiliations:** 1 Department of Civil Engineering, The University of Lahore, Lahore Campus, 1-Km Defense Road, Lahore 54590, Pakistan; muhammad.qasim@ce.uol.edu.pk; 2 Department of Mechanical Engineering, University of Engineering and Technology Lahore (Rachna Campus), Lahore 54890, Pakistan; ali.akbar@uet.edu.pk; 3 Interdisciplinary Research Center for Membranes and Water Security, King Fahd University of Petroleum & Minerals (KFUPM), Dhahran 31261, Saudi Arabia; imtiaz.khan@kfupm.edu.sa; 4 Department of Textile Engineering, National Textile University, Faislabad 37610, Pakistan; mumtaz.ali@ntu.edu.pk; 5 Department of Environmental Engineering, Daegu University, 201 Daegudae-ro, Jillyang, Gyeongsan-si 38453, Republic of Korea; lujong@daegu.ac.kr; 6 Department of Energy and Environmental Engineering, The Catholic University of Korea, 43 Jibong-ro, Bucheon-si 14662, Republic of Korea

**Keywords:** constant flux, fouling models, membrane process, water treatment

## Abstract

The goal of this study was to elucidate the interaction of complex feed solutions under modified membrane fouling models for constant flux operation. The polyvinylidene fluoride membrane (PVDF) was tested for three types of solutions containing inorganic foulants (Al, Mn, and Fe), organic foulants, and suspended solids at 0.5 mM Ca^2+^ ionic strength. The membrane’s performance was evaluated by measuring the increase in transmembrane pressure (TMP) during two different filtration scenarios: continuous filtration lasting 1 h and cyclic filtration lasting 12 min, with 3 min backwashing cycles included. Statistical analysis (linear regression results (R^2^), *p*-value) was used to verify the fouling model propagation along with the determination of the contributing constant of each fouling model. An increasing TMP percentage of 164–302%, 155–300%, and 208–378% for S1 (HA + Ca^2+^), S2 (inorganics + kaolin + Ca^2+^), and S3 (HA + inorganics + kaolin + Ca^2+^) was recorded for 1 h filtration, respectively. Furthermore, a five percent increase in irreversible resistance was noted for the S3 solution due to the strong adsorption potential of foulants for the PVDF membrane caused by the electrostatic and hydration forces of foulants. In addition to that, the participation equation elucidated the contribution of the fouling model and confirmed that complete blocking and cake layer contribution were dominant for the S1 and S3 solutions, while standard blocking was dominant for the S2 solution with a high significance ratio. Moreover, R^2^ and cyclic filtration analysis also confirmed the propagation of these fouling models. The statistical confirmation and regression results analysis of the modified model gave comparative results and satisfied the filtration mechanism and can be used for the constant flux dead filtration analysis of water treatment.

## 1. Introduction

Research has been delineated for fouling during constant pressure (CP) dead-end filtration using the blocking law, which relates the time (*t*)-dependent behavior of filtrate volume (*V*) [1].

(1) $$\frac{d^{2}t}{dV^{2}}=k\;{\left(\frac{dt}{dV}\right)}^{n}$$

Equation (1) was introduced by Hermia to address the four classical fouling models, where *n* varies according to the fouling mechanism (complete fouling (*n* = 1); intermediate fouling (*n* = 2); standard fouling (*n* = 3/2); and cake layer fouling (*n* = 0)) and k represents the fouling constant [2,3,4]. Moreover, fouling models for CP filtration were further modified by researchers for their respective fields of study. Bowen et al. modified the fouling model to identify the dominant fouling mechanism at different stages of MF membrane fouling during bovine serum albumin (BSA) filtration and tested it for different pore sizes [5]. Moreover, Ho and Zydney modified the fouling model by combining the pore blocking and cake filtration mechanisms to describe BSA protein fouling during MF [6]. However, membranes used for water and wastewater treatment are commonly operated at constant flux (CF) to meet demand [7]. In addition, limited studies were conducted to address fouling in a CF process, even though fouling laws for the time dependence of pressure (*TMP*) to maintain constant flux have been derived as Equation (2) [7].

(2) $$\frac{d^{2}t}{d{\left(TMP\right)}^{2}}=k\;{\left(\frac{dt}{d(TMP)}\right)}^{n}$$

Experimental verification of CF fouling has been limited to protein extraction and tertiary wastewater treatment for consideration of pore-blocking or incompressible cake. Therefore, limited applicability for real-world materials exists because of the increase in specific resistance to water flow with pressure [8].

Generally, membrane fouling studies were conducted using model foulants to represent the specific type of foulant mixture; for example, latex bead and silica particles in feed solution were used to represent solid particles [9]. Moreover, soybean oil emulsion, BSA, and bacteria suspension were used to address oil/water emulsion, protein, and microorganism fouling. It is worth noting that the behavior of these model foulants can vary based on several factors. Concentration, for instance, can significantly affect how foulants interact with the membrane. Likewise, properties like hydrophobicity and surface charge can influence the fouling behavior, making it essential to account for these variables in fouling studies [3,10]. To further enhance the comprehensiveness of fouling investigations, researchers have explored the use of multiple foulants in combination [11]. This approach allows them to replicate complex fouling scenarios that membranes may encounter under various operating conditions [12,13,14]. By studying the interplay of different foulants, researchers can gain insights into the multifaceted nature of membrane fouling and develop more robust strategies for fouling prevention and mitigation.

Field et al. reported the threshold and critical flux concept for yeast cell suspension and oil/water emulsion for membranes [15]. Krischner et al. also reported the threshold and critical flux for latex bead suspension and soybean oil emulsion [9], similar to another study [16]. However, the combination of inorganic foulants (Al, Mn, and Fe) with organic foulants and their impact on membrane fouling was not reported in detail [17,18,19]. In addition, fouling propagation of membranes due to Ca^2+^ and Mg^2+^ was well addressed on the basis of an interaction mechanism for HA and membranes [20,21]. Ion binding and aggregation with HA by Ca^2+^ and Mg^2+^ is considered to cause an increase in membrane fouling due to the formation of cationic complexes with HA to form a thick foulant layer on the membrane based on electrostatic interaction and shielding due to hydration layer formation during filtration [21,22,23,24]. Few or no studies were conducted to elucidate the fouling behavior of membranes for inorganics (based on electrostatic interactions and hydration) operated in CF dead mode in the presence of organic foulants and suspended solids. These inorganic foulants are also present in surface water along with Ca^2+^ and Mg^2+^ in the form of residual inorganics [19,25]. Moreover, the new methodology was introduced by using a participation (contribution) equation along with regression results (R^2^) for the propagation of the fouling model, with a significant impact on the overall process.

The current study presents a modification of Hermia’s fouling model equations specifically tailored to constant flux dead-end filtration. To assess the behavior of fouling under various conditions, equations were introduced to calculate the contribution constants of fouling models in relation to the operating flux. These assessments took into consideration different types of feed solutions and how contaminants interacted with the membrane. In this study, three distinct feed solutions were employed, comprising humic acid (HA) and various metal ions (Fe, Al, and Mn) as the organic and inorganic foulants in the feed water. The ionic strength of the solution was maintained at 0.5 mMole of Ca^2+^, representative of surface water conditions. To investigate fouling dynamics, the progression of individual fouling models, including complete fouling, intermediate fouling, standard fouling, and cake layer fouling, was rigorously evaluated through regression analysis. These evaluations were carried out during two different filtration scenarios: continuous filtration lasting 1 h and cyclic filtration involving 12 min of filtration and 3 min of backwashing. To comprehensively assess the impact of various flow rates on fouling behavior, five different flow conditions were considered: 40, 80 LMH (low-flow), 120 LMH (intermediate-flow), and 160, 200 LMH (high-flow). Furthermore, a comparative analysis of the results obtained under the aforementioned experimental conditions was conducted to gain insights into the applicability and effectiveness of the modified fouling models for water treatment purposes.

## 2. Model Development

The equations derived below are appropriate for constant flux (CF) dead-end filtration. In CF operations, shear force from the feed side pushes the foulants to be deposited on the membrane surface [26], which results in a decrease in the effective pore and tends to increase the flow through other pores to compensate for the permeate flux requirement [9,27].

The derivation below follows Hermia’s model development for constant Δ*P* dead-end filtration and recasts it for CF dead-end filtration [2]. Therefore, adjustments were made to the fouling equations to compensate for the CF operation parameters that incorporate the surface fouling mechanism in dead-end filtration, including complete fouling, intermediate fouling, standard fouling, and cake layer fouling. However, the standard fouling mechanism is not the surface fouling phenomenon found in dead-end filtration. Increasing pressure on the surface pushes the particles to be trapped in pores and adsorbed to the membrane due to association with the membrane [17]. Moreover, a schematic of the four typical fouling models is illustrated in Figure S1a–d (Supplementary Documents).

### 2.1. Complete Fouling Model

In the complete fouling model (*n* = 2), it is assumed that each particle that encounters the membrane blocks a pore without superposition, ultimately leading to a decrease in the effective membrane area [4].

Generally, Darcy’s law is used to calculate flow through a porous membrane [9]:

(3) $$Q=\frac{\Delta Pa}{µR_{m}}$$

where *Q* is the flow rate (m^3^/s), Δ*P* is the transmembrane pressure (kPa), a is the membrane effective (clean, unobstructed) surface area (m^2^), µ is the permeate viscosity (Pa.s), and *R_m_* is intrinsic membrane resistance (m^−1^).

In a CF operation, *Q* is the maintaining constant for time 0 → t. As the overall resistance throughout the process is considered to be the same, a decrease in the unobstructed surface area of the membrane results in increased pressure from the other pores to meet the CF condition. Therefore, variation in pressure through pores becomes [28]:

(4) $$\Delta P_{t}=\frac{\Delta P_{0}a_{0}}{a_{t}}$$

where initial and final conditions at time *t* are represented by subscripts 0 and *t*, respectively. The change in membrane surface area with time can be expressed as a function of volume filtered through area [2]:

(5) $$a_{t}=a_{0}-\sigma V$$

where *σ* is the blocked membrane surface area per unit filtrate volume (m^−1^). Rearranging Equation (5) and introducing the decrease in membrane filtration area through membrane surface area, the equation becomes

(6) $${\frac{da}{dt}=-\sigma \left(a_{0}-a\right)J}_{\;}$$

where (*a*_0_ − *a*) expresses the rate of decrease in unobstructed surface area due to foulant deposition on the membrane surface blocking the pores [15,29]. Integrating Equation (6) generates an expression of unobstructed area as a function of time:

(7) $${a_{t}=\frac{a_{0}}{\sigma Jt}\;(1-\exp\left(-\sigma Jt\right))}_{\;}$$

Substituting Equation (7) into Equation (4) gives Equation (8)

(8) $${\Delta P_{t}=\frac{\Delta P_{0}\;t\;K_{b}}{1-\exp\left(-K_{b}t\right)}\;}_{\;}$$

where *K_b_* is the complete fouling constant (s^−1^) defined as

(9) $${K_{b}=\sigma J}_{\;}$$

When the second term in the denominator of Equation (8), exp(−*K_b_ t*), is between 0 and 1, the model predicts a rise in Δ*P* with time, and it approaches infinity when it is equal to 1. In Hermia’s fouling model, complete fouling was a reasonable mechanism for constant Δ*P*, as all pores are blocked and the permeate flow rate declines to zero. However, in CF operations, all pores being completely fouled is not a realistic approach, as in some conditions complete fouling predicts a similar Δ*P* profile as the intermediate fouling mechanism [9]. Moreover, individual inorganic foulants alone would not contribute to complete fouling as these foulants are soluble in water and pass through the membrane pores. However, with the combination of NOMs, a complex is formed which is deposited on the membrane surface to cause complete fouling.

### 2.2. Intermediate Fouling Model

In the intermediate fouling model (*n* = 1), it was assumed that some particles partially block membrane pores and other particles settle over the blocking particles [2].

The probability of a particle depositing on a previously settled particle will be represented as the rate of change of the unobstructed membrane surface area, based on Equation (10):

(10) $${{\frac{da}{dt}=-\sigma aJ}_{\;}}_{\;}$$

The difference in area accounted for by Equation (6) and Equation (10) represents the area of particles deposited on already-blocked pores or partially blocked particles after integration, and rearranging the result in Equation (11) [2,30] produces:

(11) $$\begin{array}{c} a_{t}=K_{i}V\exp\left(-K_{i}Jt\right)\\ \mathrm{where}\;K_{i}\;=\;\sigma \end{array}$$

where *K_i_* is the constant for intermediate fouling (m^−1^). Thus, substitution of Equation (11) into Equation (4) results in:

(12) $${{\Delta P_{t}=\frac{\Delta P_{0}}{\exp\left(-K_{i}Jt\right)}}_{\;}}_{\;}$$

Equation (12) reveals no inconsistencies like the complete fouling model; the denominator of the equation remains positive with a max value of one. The equation predicts the initial increase in Δ*P* with a constant rate for a long time, and the plateau of Δ*P* results confirms the decreasing probability of a foulant particle blocking an open pore as more and more pores are blocked.

### 2.3. Standard Fouling Model

In the standard fouling model (*n* = 3/2), particles smaller than the pores of the membrane settle on the inner wall through the adsorption property and cause pore constriction. Standard pore blocking assumes blocking inside the membrane pores rather than on the surface [31].

Membranes are considered to have laminar flow similar to the flow through capillary tubes with radius (r) (assuming that the membrane has cylindrical pores with tortuosity = one); thus, the Hagen–Poiseuille law is used to describe the flux, as illustrated in Equation (13) [32]:

(13) $${{J_{0}=\frac{\varepsilon r_{o}^{2}(\Delta P_{0})}{8\tau µ\Delta x}}_{\;}}_{\;}$$

where *ε* is the porosity (dimensionless), *r*_0_ is the initial pore radius (m), Δ*P*_0_ is the transmembrane pressure (KPa), *τ* is the pore tortuosity factor (dimensionless), and Δ*x* is the membrane thickness (m).

As we are working with constant flux, *J*_0_ = *J_t_* for time 0 → t with an increase in Δ*P*_0_ to Δ*P_t_* and *r*_0_ to *r_t_*. Thus, Equation (13) becomes for time *t*

(14) $${{J_{t}=\frac{\varepsilon r_{t}^{2}(\Delta P_{t})}{8µ\tau \Delta x}}_{\;}}_{\;}$$

By comparing and rearranging the Equations (13) and (14) results,

(15) $${{\Delta P_{t}={\left(\frac{r_{0}}{r_{t}}\right)}^{2}\Delta P_{0}}_{\;}}_{\;}$$

Following Hermia’s solid mass balance yields [2]:

(16) $${{N\pi \;\left(r_{0}^{2}-r_{t}^{2}\right)∆x=CV}_{\;}}_{\;}$$

where *C* is the volume of particles deposited per unit volume of filtrate. Rearranging Equation (16) and the desired result ratio to Equation (17),

(17) $${{{\left(\frac{r_{t}}{r_{0}}\right)}^{2}=\left(1-\frac{CV}{N\pi r_{0}^{2}∆x}\right)}_{\;}}_{\;}$$

where the constant for standard fouling *K_s_* (m^−3^) is defined as

(18) $${{K_{s}=\frac{C}{N\pi ∆xr_{0}^{2}}}_{\;}}_{\;}$$

Substituting Equation (17) into Equation (15) and rearranging results in

(19) $${∆P_{t}=\frac{∆P_{0}}{\left(1-K_{s}a_{0}Jt\right)}}_{\;}$$

The standard fouling mentioned in Equation (19) is applicable to CF filtration. Consequently, *K_s_a*_0_*Jt* is always positive and ranges between 0 and 1, and thus the denominator remains less than 1 while predicting the Δ*P* for standard fouling. A similar correction should be made for standard fouling as with complete fouling while breakdown occurs with the blocking of all pores, as the pore radius decreases from inside will increase the velocity to maintain the constant flux. The increase in velocity is responsible for extra shear, resulting in the increase in Δ*P* in the standard fouling model. Another assumption is that standard fouling is predominant when the particle size is smaller than the pore diameter, which will cause a decrease in the effective radius of the pore and cause an increase in Δ*P*.

### 2.4. Cake Layer Fouling Model

In the cake layer model (*n* = 0), the clogging of membrane pores by the deposition of particles on the membrane forms a cake, leading to a decrease in filtrate volume [2]. Cake layer resistance is expressed as the summation of clean membrane resistance and the resistance of foulant layer deposition, as mentioned above [15,33]:

(20) $${R_{t}=R_{0}+\frac{\alpha W}{a_{0}}}_{\;}$$

where α is the cake-specific resistance (m/kg) and *W* is the cake mass (kg) from the mass balance on the cake [9,29]:

(21) $${W=\frac{V\gamma s}{\left(1-ms\right)}}_{\;}$$

where *γ* is the filtrate density (kg/m^3^), s is the mass fraction of the fouling solution, and m is the wet-to-dry cake mass ratio. Substituting Equation (21) into Equation (20)**,** the overall resistance of the filter media becomes:

(22) $${R_{t}=R_{0}\;\left(1+K_{gl}Jt\right)}_{\;}$$

where *K_gl_* in Equation (22) represents the constant for cake layer filtration (m^−1^), which is equal to

(23) $${K_{gl}=\frac{\alpha \gamma s}{\left(1-ms\right)R_{0}}}_{\;}$$

The substitution of Equation (22) into Equation (3) with rearrangement results in:

(24) $${{\Delta P}_{t}={\Delta P}_{0}\;\left(1+K_{gl}Jt\right)}_{\;}$$

Equation (24) is the final version of the cake layer filtration equation and shows a linear relationship with t, similar to the resulting equation mentioned elsewhere [9]. There is a contradiction in assessing the cake layer filtration as it is assumed that the cake covers the membrane surface completely, which is not entirely true for a short period of filtration. However, for long-term filtration, the dominant fouling mechanism would be cake layer fouling.

## 3. Materials and Methods

### 3.1. Materials

All chemicals were of analytical grade, unless otherwise mentioned. Twelve percent sodium hypochlorite (NaOCl; CAS number: 7681-52-9) was provided by UNI Chemicals and Co., Ltd. (Ansan-si, Republic of Korea) Ferric chloride anhydrous (FeCl_3_; CAS number: 7705-08-0), anhydrous calcium chloride (CaCl_2_; CAS number: 7440-70-2), and alum (KAl [SO₄] [Chellam, 2006 #229;Suarez, 2000 #255;Field, 1995 #308]₂·12H₂O; CAS number: 10043-67-1) were purchased from Daejung Chemical Reagents and Metals Co., Ltd. (Siheung, Republic of Korea). Humic acid (HA) and kaolin were purchased from Sigma-Aldrich Co., Ltd. (St。, Louis, MO, USA). Mn (II) sulfate (CAS number: 10034-96-5) was obtained from Junsei Chemical Co., Ltd. (Tokyo, Japan). Hollow fiber PVDF membranes (microfiltration membrane) were purchased from TORAY Chemical Ltd., (Tokyo, Japan) and membrane characteristics and operational conditions were elucidated from Table S1. Membranes were soaked in deionized (DI) water for 24 h before use and were stored and rinsed in DI water after use to prevent desiccation.

### 3.2. Analytical Techniques

Membrane surface morphology was examined by field emission scanning electron microscopy (FE-SEM, Nano.,S-4800, Hitachi, Tokyo, Japan). Moreover, the interaction of inorganic contaminants with the membrane was examined by confirming the presence of foulant with energy dispersive spectroscopy (EDS, Quanta-200, FEI, Hillsboro, OR, USA). A zetasizer (Nano-ZS90, Malvern Instruments, Ltd., Malvern, UK) was used to measure the particle size in solution.

### 3.3. Membrane Exposure and Fouling Analysis

Membranes were exposed to synthetic feed solution, in which NOMs and inorganic foulants were prepared in deionized water. The concentration and characteristics of feed water have been reported elsewhere [17]. Furthermore, the ionic strength of the feed water was controlled by the addition of 0.5 mM Ca^2+^ using CaCl_2_. The feed solution characteristics used for the dead-end constant flux (CF) operation are illustrated in Table S2.

CF filtration experiments were conducted for 40–200 LMH, and the experimental setup is shown in Figure S2 (Supplementary Documents). The permeate flow was controlled by a peristaltic pump to ensure the desired flux and rise in TMP was recorded for each case. Furthermore, the experimental setup was divided into two types of filtration: (a) continuous filtration, including each cycle of 1 h, and (b) periodic filtration, 3 cycles of 12 min filtration and 3 min of backwashing. The reason for using different filtration times was to analyze the impact of a long filtration cycle and periodic filtration cycle on the rise in TMP. The filtration procedure comprised 15 min of filtration of DI water at CF and then replacing the DI water with feed water. A little change in TMP was observed at the start due to the variation of the mass transfer rate, which was normalized by controlling the feed pressure. The data of the TMP variation were recorded with the LABVIEW program. After filtration for 1 h, a backwash for 3 min was performed. Moreover, after filtration conditions for the membrane were tested for 5 CF conditions, the membrane was thoroughly cleaned with DI for SEM and EDS analysis. Division of flux was performed on the basis of checking the modified model of CF dead-end filtration for low-flow (40, 80 LMH), intermediate-flow (120 LMH), and high-flow conditions (160, 200 LMH). Evaluating the participation level of the fouling model during filtration, the participation equations were introduced to reveal the increasing and decreasing behavior of the fouling models throughout the process. Linear regression was applied to elucidate variations in the predicted model and the actual experimental values of the complete, intermediate, standard, and cake layer modified fouling models and to estimate the variation in membrane fouling model contribution and elucidate its significance through Equation (25) with respect to membrane flux.

(25) $${TMP=\beta_{b}TMP_{b}+\beta_{i}TMP_{i}+\beta_{s}TMP_{s}+\beta_{gl}TMP_{gl}}_{\;}$$

where, *β_b_*, *β_i_*, *β_s_*, and *β_gl_* represent the contribution constants for complete fouling, intermediate fouling, standard fouling, and cake layer fouling, respectively. Moreover, *TMP_b_*, *TMP_i_*, *TMP_s_*, and *TMP_gl_* represent the respective predicted transmembrane pressure through modified fouling models by regression result analysis.

The membrane fouling ratio was calculated using Equations (26)–(28):

(26) $${R_{m}=\frac{TMP_{0}}{\mu J}}_{\;}$$

(27) $${R_{f}=\frac{TMP_{f}}{\mu J}}_{\;}=R_{ir}+R_{re}$$

(28) $${R_{ir}=\frac{TMP_{1}-TMP_{0}}{\mu J}}_{\;}$$

where *R_m_*, *R_f_*, *R_ir_*, and *R_re_* are the membrane intrinsic resistance, fouling resistance, irreversible resistance, and reversible resistance in (m^−1^), and *TMP*_0_, *TMP_f_*, and *TMP*_1_ are the clean water transmembrane pressure, filtrate pressure, and clean water pressure after backwashing.

## 4. Results and Discussion

### 4.1. Membrane Constant Flux Dead-End Fouling Experiment

**Figure 1:**
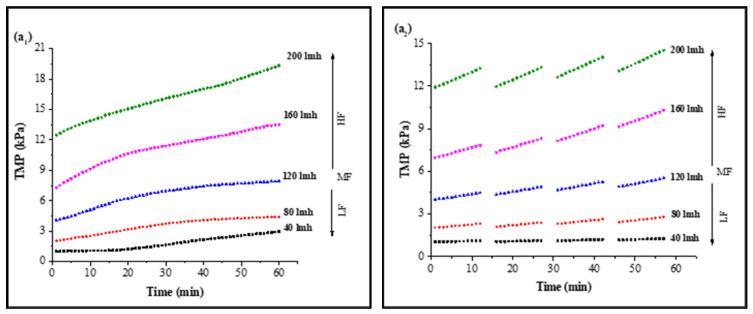

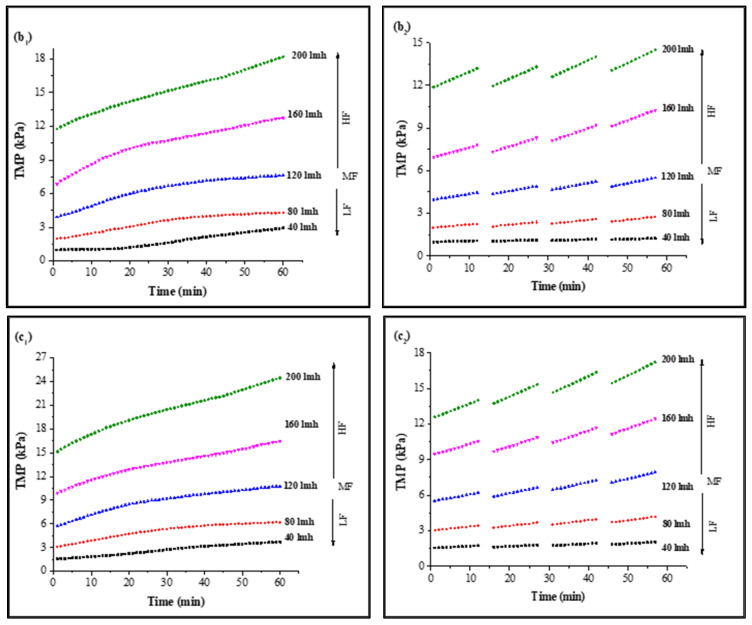
Influence of filtration time on TMP in constant flux dead-end fouling experiments: (**a**): S1; (**b**): S2; (**c**): S3 (subscripts one and two indicate continuous and cyclic filtration, respectively) [LF: low flux; MF: medium flux; HF: high flux].

In CF dead-end filtration experiments, TMP was recorded as a function of time to minimize the effect of flux variation on model parameters and membrane sample selection. Membrane samples selected for analysis had a clean water TMP starting from 0.980 ± 0.03 kPa to 11.767 ± 0.7 kPa for 40 LMH to 200 LMH by the flux stepping method, as shown in Figure S3. Results for continuous filtration and cyclic filtration are illustrated in Figure 1. Figure 1(a_1_,a_2_) shows the increase in TMP of a PVDF membrane tested for feed solution containing HA along with 0.5 mMole Ca^2+^ as foulants for 1 h filtration and 12 min filtration cycles. Increases of 302%, 214%, 194%, 185%, and 164% of TMP for 1 h continuous filtration and 128%, 135%, 135%, 141%, and 126% for 12 min cyclic filtration were observed under 40, 80, 120, 160, and 200 LMH, respectively, at the end of S1 solution filtration. Similar increases of 300%, 209%, 187%, 175%, and 155% for 1 h filtration and 126%, 133%, 134%, 141%, and 121% for cyclic filtration for S2 solution testing are illustrated in Figure 1(b_1_,b_2_)**,** and the TMP increases of 378%, 302%, 259%, 226%, and 208% for 1 h filtration and 209%, 203%, 194%, 170%, and 151% for cyclic filtration for S3 solution testing under 40, 80, 120, 160, and 200 LMH CF conditions, respectively, are shown in Figure 1(c_1_,c_2_).

The results illustrated in Figure 1 confirm that the increase in operating flux led to a rapid increase in membrane TMP. A lower increase in TMP was observed for low-flow conditions compared to medium- and high-flow conditions. However, in cyclic filtration, the increase in TMP was lower compared to continuous filtration because the foulants were not allowed to completely block the membrane and deposit on the surface as in continuous filtration. Furthermore, the fouling layer was frequently disturbed by backwashing, which was responsible for the lower increase in membrane TMP during the cyclic filtration of the membrane regardless of operating flux.

The fouling solution also played a significant role in membrane performance and increase in TMP under different operating conditions. Solution S1 containing HA and Ca^2+^ as foulants generated more fouling compared to the S2 solution containing inorganic foulants (Al, Fe, and Mn) along with Ca^2+^, as the PVDF membrane has a strong potential to adsorb HA due to strong hydrogen bonding efficacy between the PVDF polymers and hydroxyl groups of HA molecules [34]. Moreover, the presence of Ca^2+^ exacerbated the fouling role because of the formation of intermolecular complexes that lead to the development of a compacted fouling layer on the membrane surface. However, the role of Ca^2+^ in the presence of inorganic foulants was different than with HA molecules. The TMP increased slowly in the presence of inorganic base salts, which required a longer filtration time to reach the TMP level of the S1 solution since inorganic salts passed through the microfiltration membrane pores and attached to the pore wall. However, a small amount of these inorganics was separated via agglomeration with suspended particles due to their flocs-forming tendency [18]. Furthermore, few synergistic effects occurred in the presence of these inorganic foulants that led to cake layer fouling, and their role in increased fouling was further elevated in the presence of HA, as illustrated through the results of the S3 solution. In addition, the more rapid increase in TMP with the S3 solution filtration was due to the complex mixture of inorganic and organic foulants in the presence of suspended particles and Ca^2+^.

**Figure 2:**
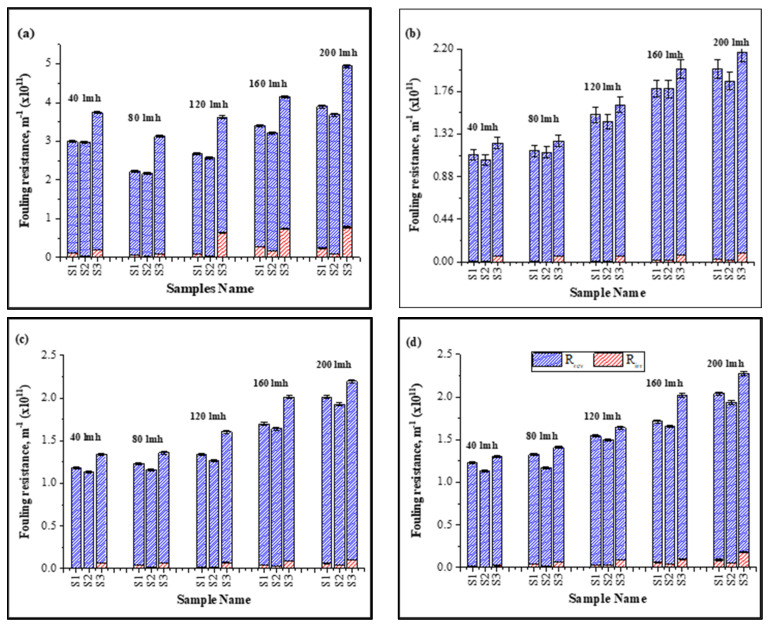
Influence of continuous and cyclic filtration in fouling resistance under different fluxes: (**a**): 1 h filtration; (**b**): first cycle; (**c**): second cycle; (**d**); third cycle of filtration.

Inorganic foulants (cations) contributed to the increase in specific fouling resistance shown in Figure 2 due to an increase in the electrostatic forces and hydration forces of membrane–foulant interactions, forming a thick layer on the membrane surface [20,35]. Moreover, the bridging effect of these inorganic foulants with HA and the membrane was strong enough that they were not removed efficiently by hydraulic cleaning, resulting in an increase in irreversible specific resistance, as shown in Figure 2.

With the increase in operating flux, the ratio of irreversible resistance increased as compared to the low-flux conditions. At high flux, particles more rapidly deposit on the membrane surface and the membrane-effective area decreases, exerting more pressure to meet the CF conditions. A thicker foulant layer develops and increases the membrane–foulant interaction due to the bridging effect, resulting in an increase in irreversible resistance [21]. Results shown in Figure 2 illustrate the increase in irreversible resistance (×10^11^ m^−1^) from 0.11 ± 0.018–0.242 ± 0.021 for S1, 0.0424 ± 0.001–0.19 ± 0.002 for S2, and 0.194 ± 0.05–0.784 ± 0.04 for S3 for 1 h continuous filtration under a constant flux of 40–200 LMH, respectively. Similar results were observed for the cyclic filtration; however, the increase in irreversible resistance in cyclic filtration was lower compared to continuous filtration due to the frequent backwashing of the membrane after 12 min. Moreover, most of the fouling resistance that contributed to the rise in TMP was reversible and its contribution was approximately >95% of the total fouling resistance in all cases.

### 4.2. Verification of Fouling Model and Analysis of Fouling Behavior

#### 4.2.1. Fouling Solution S1 (HA + Ca^2+^)

**Figure 3:**
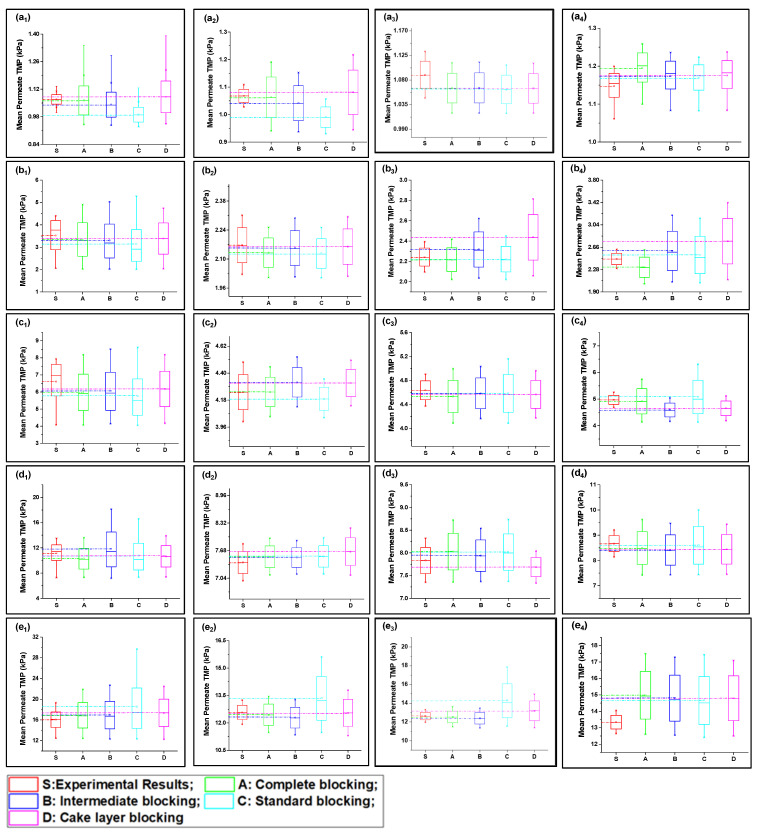
Mean variation in experimental results of HA + Ca^2+^ filtration (S1) in comparison with predicted results from four fouling models at different constant flux operations: (**a**) 40 lmh, (**b**) 80 lmh, (**c**) 120 lmh, (**d**) 160 lmh, and (**e**) 200 lmh [subscripts 1, 2, 3, and 4 show the 1 h continuous filtration and cycle 1, cycle 2, and cycle 3 of cyclic filtration, respectively].

Fitted curves of the newly developed model values are shown in Figures S4–S23 (Supplementary Documents) for 40, 80, 120, 160, and 200 LMH flows. The regression results and fouling constants for these modified fouling models are summarized in Table 1. The variation in mean values shown in Figure 3 elucidates that the complete fouling model was the main reason for the increase in TMP pressure at low flux; however, the fouling mechanism shifted to cake layer fouling for high flux for S1 filtration. Moreover, the standard fouling showed a modest role in the fouling mechanism, as illustrated through Figure 3(a_1_–e_4_). The mean values of the complete and cake layer fouling models better corresponded with the experimental results compared to standard fouling. The reason for the lower contribution of standard fouling in constant filtration of S1 fouling conditions was due to the larger particle size as compared to the membrane nominal pore size. Moreover, the phenomenon of the particle removal mechanism from inside pores accounts for the filtration process proceeding as the pore radius decreases and the filtrate velocity increases to maintain the constant flux, which leads to the removal of previously settled particles due to the increase in shear forces instead [19].

At low-flow conditions, the fouling contribution was complete fouling, and intermediate fouling would be the second dominant fouling mechanism, confirming the fouling theory as shown in Figure 3(a_1_,b_1_). Interestingly, with the increase in permeate flux, the fouling mechanism mode shifted from complete fouling to cake layer fouling, as illustrated through Figure 3(c_1_,d_1_,e_1_). As particles were rapidly deposited and blocked the membrane pores, further incoming particles deposited very rapidly on the previously settled particles to increase the thickness of the cake layer; thus, dense caking was observed with the increase in permeate flux. R^2^ values also confirmed that the complete blocking model followed by the intermediate and cake layer fouling better corresponded to the fouling mechanism as compared to the standard fouling model in the S1 solution in the constant flux operation. In addition, different behavior was observed for the constant pressure operation, as uniform thickness of the cake layer was observed due to the non-availability of particles to deposit on the membrane, leading to reduced flux. Moreover, the cyclic filtration results shown in Figure 3a–e with subscripts 2, 3, and 4 elucidated the variation of the fouling mechanism under small cycles. The results for the S1 solution illustrated that the cake layer formation was delayed in the initial stages because of the disturbance of the process. However, at the end of the third cycle, the cake layer fouling start dominated, as confirmed through the R^2^. Moreover, fouling propagation would be similar to the 1 h filtration with the increase in permeate flux.

#### 4.2.2. Fouling Solution S2 (Inorganic Foulants + Turbidity + Ca^2+^)

**Figure 4:**
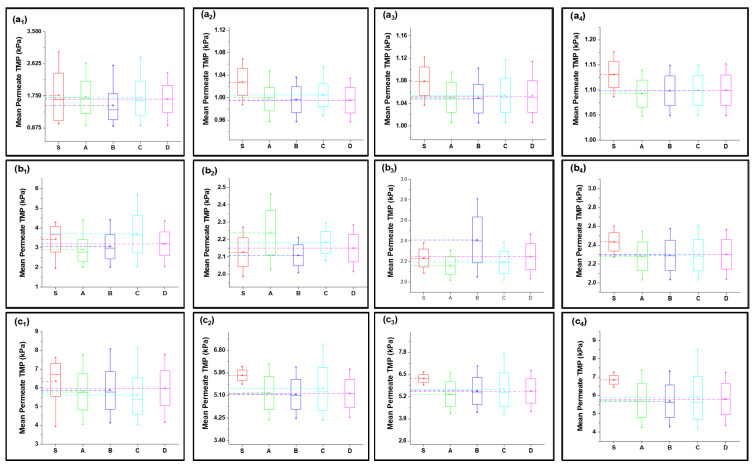

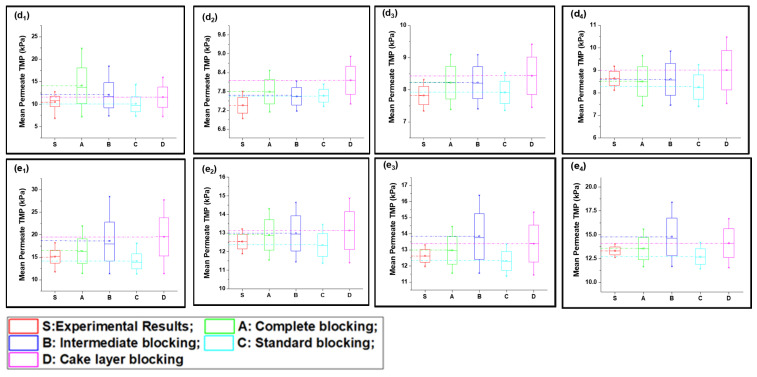
Mean variation in experimental results of inorganic foulant + turbidity + Ca^2+^ filtration (S2) in comparison with predicted results from four fouling models at different constant flux operations: (**a**) 40 lmh, (**b**) 80 lmh, (**c**) 120 lmh, (**d**) 160 lmh, and (**e**) 200 lmh [subscripts 1, 2, 3, and 4 show the 1 h continuous filtration and cycle 1, cycle 2, and cycle 3 of cyclic filtration, respectively].

The S2 solution possessed a different fouling mechanism, with predominant standard fouling of the membrane along with the complete fouling model, as shown in Figure 3(a_1_–e_4_). The presence of inorganic foulants in feed solution played the role of charge neutralization and decreased the electronegativity of suspended particles, allowing them to associate more closely and leading to the agglomeration and formation of suspended flocs. Fe^3+^ played a significant role in this process as it has poor potential to neutralize as compared to Al^3+^ [36]. In addition, the particle size of agglomerated flocs was less than the S1 particles and the majority of foulants passed through the membrane and did not settle on previously settled particles, which led to poor correspondence with the cake layer and an intermediate fouling mechanism at low-flow conditions. Moreover, with the increase in pressure, the foulants were squeezed through the membrane pore and adsorbed to the pore wall. This phenomenon was more observed in low-flow conditions and the mean values more corresponded with the complete blocking and standard blocking, as shown in Figure 4(a_1_,b_1_). The tendency of pressure increase was less compared to the S1 feed solution. In addition, in medium-flow and high-flow conditions, as shown in Figure 4(c_1_–e_1_), the particles passed through pores and deposited on membrane surfaces, causing the development of cake layer and intermediate blocking. However, the cake layer was not thick enough compared to the S1 solution, and it was removed efficiently with backwashing as elucidated through cyclic filtration, as shown in Figure 4c–e with subscripts 2, 3, and 4. Mean variation results confirmed that the standard blocking would be the prominent fouling model along with complete fouling at low-flow conditions; intermediate fouling and cake layer played trifling roles, and vice versa for high-flow conditions. This phenomenon was more clearly observed in cyclic filtration, and the increase in fouling model dominance was elucidated. The R^2^ results summarized in Table 1 also confirm the dominance of standard fouling in S2 solution filtration and decrease with the increase in flux.

#### 4.2.3. Fouling Solution S3 (HA + Inorganic Foulants + Turbidity + Ca^2+^)

**Figure 5:**
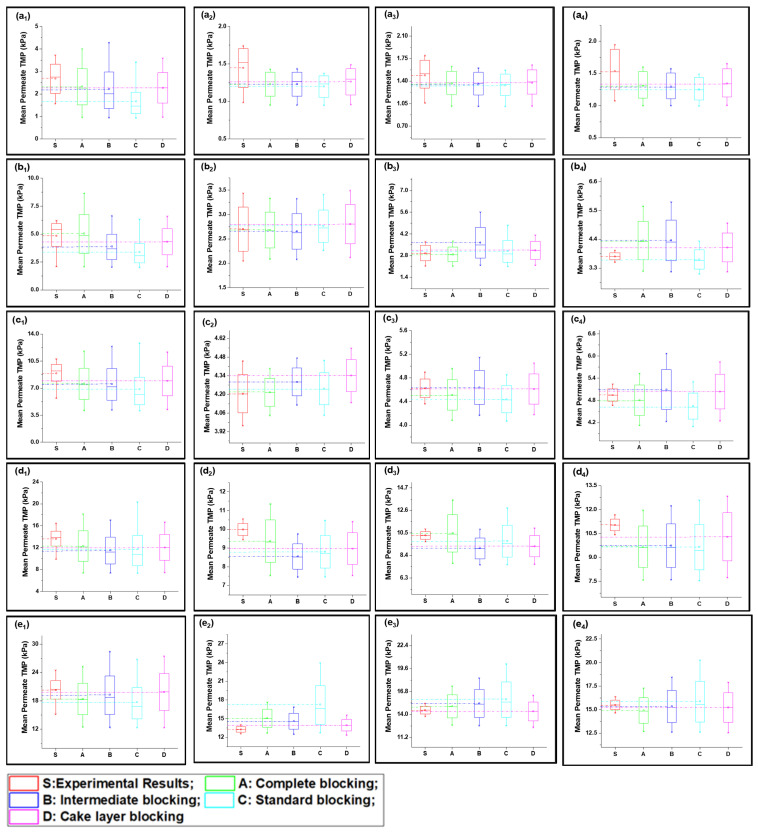
Mean variation in experimental results of HA + inorganic foulants + turbidity + Ca^2+^ filtration (S3) in comparison with predicted results from four fouling models at different constant flux operations: (**a**) 40 lmh, (**b**) 80 lmh, (**c**) 120 lmh, (**d**) 160 lmh, and (**e**) 200 lmh [subscripts 1, 2, 3, and 4 show the 1 h continuous filtration and cycle 1, cycle 2, and cycle 3 of cyclic filtration, respectively].

The mean variation results for the complex mixture containing HA and inorganic foulants in the presence of the suspended particles and Ca^2+^ are summarized in Figure 5. The results elucidated in Figure 5(a_1_–e_1_) show that the complete fouling model along with cake layer fouling were dominant for fouling propagation during 1 h continuous filtration. Moreover, standard blocking and intermediate fouling played a trifling role in high-flow conditions. Initially, at low-flow conditions the complete blocking and cake layer propagate equally, but with the increase in flux, the thick cake layer forms due to the deposition of the complex mixture, and the mean variation results shift more toward the cake layer fouling. Moreover, the R^2^ results shown in Table 1 also confirm the dominance of the cake layer fouling model in high-flow conditions and the later stages of cyclic filtration. The thick cake layer formation during the filtration of the complex mixture is produced by the increased role of the charge neutralization of HA and suspended particles due to the combined presence of inorganic salts and Ca^2+^ in the case of the S3 solution [36]. Electrostatic forces due to electrostatic shielding effects control the formation of a hydration layer around HA by Ca^2+^ and other inorganic foulants [37]. Moreover, Mn^2+^ and Ca^2+^ showed similar behavior, which might be due to their having the same water bond time (≈10^−8^ s), while Al^3+^ has 0.1 s, which is 10^7^ times longer [38,39]. Thus, the hydration layer formed by the electrostatic force of interaction would cause bonding with agglomerated flocs to form the hydrated complex. This hydrated complex formed a very dense cake layer due to a stronger bridging impact from the interaction of foulants with the membranes compared to the S1 and S2 solutions [21,40], which caused the rapid increase in TMP for S3 compared to the other foulant solutions. Moreover, the fouling model propagation in cyclic filtration is shown in Figure 4a–e with subscripts 2, 3, and 4, confirming that the propagation of complete fouling and cake layer fouling and the standard fouling role were due to the adsorption of foulants due to the increase in TMP, which tends to allow the particles to pass through the membrane and allow their deposit on the membrane pore wall.

**Figure 6:**
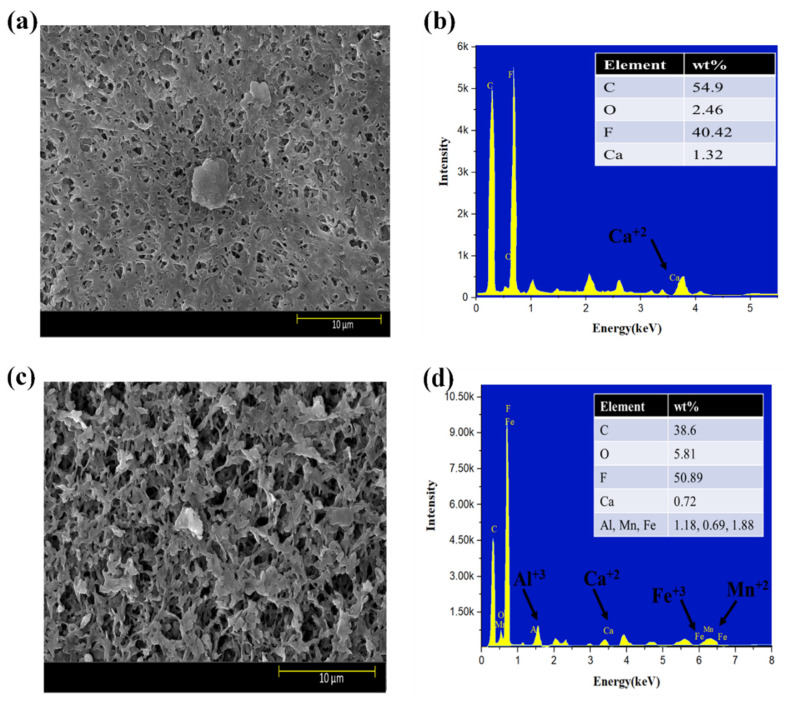

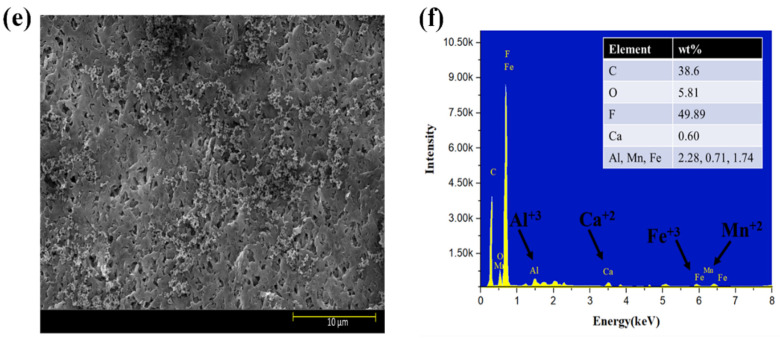
SEM and EDS analysis of exposed PVDF membranes tested for feed solution ((**a**,**b**): S1 solution; (**c**,**d**): S2 solution; (**e**,**f**): S3 solution).

Moreover, the surface morphology of membranes tested for three feed solutions at five different flux conditions are shown in Figure 6a–f. The results indicated that HA + Ca^2+^ tested membranes had a very dense cake layer compared to the S2 solution, confirming that DI water cleaning was not sufficient to remove the HA + Ca^2+^ complex adsorbed to the membrane (Figure 6a); however, DI cleaning significantly removed the deposited foulant from the S2 tested membrane (Figure 6c). The results also confirmed that the S3 tested membrane had a cake layer thoroughly distributed along with the presence of agglomerated foulants mainly caused by Fe (Figure 6e). The presence of inorganic foulants was confirmed by EDS analysis (Figure 6b,d,f). An increase in the % of Al^3+^ was observed for the S3 tested membrane compared to the S2 membrane; however, few changes in Mn^2+^ and Fe^3+^% were observed, and the presence of Ca^2+^ decreased from 0.72% to 0.60%.

SEM–EDS analysis of the membranes strengthened our observation that Al^3+^ plays a significant role in the electrostatic shielding effect compared to Ca^2+^. The formation of the cake layer in the presence of S1 was mainly due to the bridging effect of the complex of HA with Ca^2+^. However, the increase in Al^3+^ from 1.18% to 2.28% compared to Ca^2+^ (0.72–0.60%) for the S2 and S3 solutions shows the more evident results of the Al^3+^ contribution to a hydration layer when it encounters HA in the presence of Ca^2+^ and confirms the formation of a hydration layer, as explained in the earlier section. Moreover, the comparative results with the literature are elucidated in Table S3.

### 4.3. Statistical Analysis of Fouling Model Participation

A statistical analysis of the modified fouling model to find the participation of each fouling model under different fouling conditions is summarized in Table 1. Moreover, the regression results (R^2^) were also used to confirm the observation made through the statistical analysis. The results of the participation coefficient elucidated that the complete blocking model shows dominated behavior along with the cake layer fouling model for the S1 solution. The contribution coefficient of complete blocking (βb) increased from 3.09–7.95 and the cake layer fouling (βgl) from −2.09–7.83 at 40–120 lmh constant flux filtration with a high significance level (*p* < 0.05). Moreover, the standard fouling behaviors were decreased due to the lower deposition of the foulant to the wall, resulting in a decrease in the contribution coefficient from 2.01–−0.21 with a high significance level (*p* < 0.05). Furthermore, the regression results also provide statistical confirmation of the fouling model equations by following the Hermia fouling model results explained in a previous study [4]. The statistical results of the cyclic filtration also strengthen the observation while having a non-significance ratio (*p* > 0.05) in cases of low-flux conditions of S1 filtration.

The regression results of the S2 solution show that standard blocking shows the dominant fouling model along with the complete blocking and cake layer fouling. Moreover, the statistical results also confirm that the contribution constants of standard blocking (βs) increased from 2.05–8.34 along with the common significance ratio and the increase in cake layer contribution by flux variation. However, complete blocking and intermediate blocking coefficients (βi) have mixed values, with decreases in contributions, by having high significance ratios (*p* < 0.05). This might be due to the greater adsorption of the inorganic foulants to the wall as explained in Section 4.2. and the deposition of suspended particles as non-deformable particles on the surface of the membrane as a cake layer.

From the results in Section 4.2.3, it is evident that the S3 solution has a complex mixture of foulants that have a greater impact on membrane fouling propagation compared to S1 and S2, which leads to a higher fouling constant with respect to the increase in flux. The bridging effects of Ca^2+^ in the presence of HA show a more severe increase in membrane fouling potential for S1 [21]. Furthermore, this effect significantly increased the contribution constants of the fouling models in CF dead-end filtration of the S3 solution due to the presence of other inorganic foulants (Al^3+^, Mn^2+^). However, the agglomeration of inorganic salts played a minor role in individual fouling propagation but was exacerbated in the presence of HA by increasing in hydrostatic and electrostatic forces of interaction (agglomeration, S2 + bridging effects, S1). The statistical results also confirmed that βb decreased with the increasing flux from 40–200 LMH, with a contribution variation of 5.31–−7.64. However, the βgl increased from 4.31–8.64 at the same condition with a non–significance level of *p* = 0.091 in the initial stages to a high significance level of *p* = 2.08 × 10^−36^ from 40–200 LMH, respectively. Moreover, the parametric results show that βs increased from 4.19 to 9.89 with a statistically non-significant ratio (*p* = 0.10) to a significant ratio (*p* < 0.05). In addition to that, a decrease in βi was observed, similar to the βb, suggesting that in complex mixture filtration the cake layer fouling would be the dominant fouling model. Moreover, the cyclic filtration results also confirmed the propagation of these fouling models with a statistically significant ratio (*p* < 0.05).

## 5. Conclusions

The objective of this study was to adapt fouling models specifically for constant flux dead-end filtration, a crucial process involving microfiltration membranes used in water treatment. Additionally, the study aimed to shed light on the fouling behavior of these membranes when exposed to different fouling solutions. The findings of the research revealed some noteworthy observations. Firstly, it was observed that in the case of the S1 solution, which involved HA and Ca^2+^, the dominant fouling mechanism was the bridging of HA with Ca^2+^. This mechanism resulted in a more substantial increase in TMP when compared to the agglomeration of inorganic particles with Ca^2+^ and suspended solids. In more complex mixtures like the S3 solution, Al^3+^ played a multifaceted role. It contributed to the formation of a hydration complex with HA through the bridging mechanism, alongside Ca^2+^ and Mn^2+^. Simultaneously, it participated in the agglomeration of suspended particles. This multifunctional role of Al^3+^ in S3 led to a rapid increase in TMP and the development of a more intricate fouling layer, ultimately resulting in a higher level of irreversible resistance compared to other fouling solutions. Furthermore, it was observed that as the operating flux increased, irreversible resistance also increased. This phenomenon was attributed to the deposition of a thick layer of foulant on the membrane, reducing the effective filtration area and necessitating a rapid TMP increase to maintain the desired flux conditions. Regression analysis was employed to validate the fouling model. The results confirmed that in the case of the S1 and S3 solutions, complete fouling and cake layer fouling were the dominant mechanisms, and their contributions increased with higher operating flux. On the other hand, the S2 solution exhibited a standard fouling contribution primarily due to the adsorption of particles on the pore walls of the membrane. Statistical analysis of the data indicated variations in the respective contribution constants, along with their significance ratio variations. These findings emphasized the effectiveness of the modified fouling model for low-pressure membrane filtration and underscored the utility of participation equations for better representation of combined fouling mechanisms.

## Figures and Tables

**Table 1:** Regression analysis of modified fouling models for different feed water solutions at various constant flux conditions.

Models	Fitting Parameters	S1 Solution				S2 Solution				S3 Solution			
		Complete Blocking (K_b_) × 10^−4^	Intermediate Blocking (K_i_)	Standard Blocking (K_s_)	Cake Layer Blocking (K_gl_)	Complete Blocking (K_b_)	Intermediate Blocking (K_i_)	Standard Blocking (K_s_)	Cake Layer Blocking (K_gl_)	Complete Blocking (K_b_)	Intermediate Blocking (K_i_)	Standard Blocking (K_s_)	Cake Layer Blocking (K_gl_)
***40 lmh***													
1 h continuous filtration	K R^2^	7.52 0.9868	31.3 0.9708	95.4 0.9276	44.9 0.9146	7.40 0.9392	30.9 0.9658	94.9 0.9534	44.1 0.8966	11.9 0.7217	42.7 0.6596	107.7 0.6133	80.3 0.8593
Participation equation		TMP = 3.09TMP_b_ − 1.75TMP_i_ + 2.01TMPs − 2.93TMP_gl_				TMP = 8.19TMP_b_ − 2.27TMP_i_ + 2.05TMPs − 3.34TMP_gl_				TMP = 5.31TMP_b_ − 6.14TMP_i_ + 4.19 TMPs + 4.31TMP_gl_			
*p*-value		5.74 × 10^−13^	1.74 × 10^−07^	0.018	3.25 × 10^−17^	1.07 × 10^−12^	2.47 × 10^−7^	0.002	5.25 × 10^−17^	0.089	0.097	0.10	0.091
Cycle 1	K R^2^	3.77 0.5496	19.5 0.3928	88.5 0.5325	18.4 0.5453	2.25 0.9935	12.1 0.9494	56.8 0.9987	11.4 0.9295	6.5 0.9182	32.4 0.8893	83.2 0.7692	52.4 0.9709
Participation equation		TMP = −6.51TMP_b_ +6.49TMP_i_ − 1.49TMPs + 1.85TMP_gl_				TMP = −0.74TMP_b_ + 5.50TMP_i_ + 0.98TMPs − 2.28TMP_gl_				TMP = 1.86TMP_b_ − 2.41TMP_i_ + 3.15TMPs + 1.31TMP_gl_			
*p*-value		9 × 10^−4^	0.057	0.069	1.5 × 10^−4^	0.060	0.054	0.052	0.0597	0.031	0.036	0.039	0.034
Cycle 2	K R^2^	4.56 0.6248	23.4 0.5367	89.6 0.5468	22.8 0.5127	4.22 0.7832	21.6 0.7138	85.3 0.7687	21.0 0.7418	9.7 0.9142	35.3 0.8654	94.4 0.6734	67.3 0.9527
Participation equation		TMP = 0.47TMP_b_ − 0.19TMP_i_ − 0.18TMPs + 0.25TMP_gl_				TMP = 0.55TMP_b_ − 0.23TMP_i_ + 1.18TMPs − 2.26TMP_gl_				TMP = 3.31TMP_b_ − 3.21TMP_i_ + 3.19TMPs + 2.42TMP_gl_			
*p*-value		0.85	0.92	0.91	0.046	0.013	0.96	0.019	0.046	0.012	0.013	0.013	0.010
Cycle 3	K R^2^	6.98 0.6327	29.8 0.5108	92.5 0.6987	34.8 0.5837	6.13 0.6132	30.6 0.6944	89.3 0.6237	30.7 0.9872	10.1 0.9806	41.6 0.9586	102.3 0.8456	72.5 0.9834
Participation equation		TMP = 0.61TMP_b_ − 0.01TMP_i_ + 0.06TMPs + 0.20TMP_gl_				TMP = −1.55TMP_b_ + 2.21TMP_i_ + 1.15TMPs − 2.67TMP_gl_				TMP = 4.74TMP_b_ − 4.21TMP_i_ + 3.24TMPs + 3.07TMP_gl_			
*p*-value		9.4 × 10^−3^	0.012	0.014	4.1 × 10^−3^	0.14	0.11	0.115	0.021	6.6 × 10^−3^	4.9 × 10^−3^	4.3 × 10^−3^	6.1 × 10^−3^
***80 lmh***													
1 h continuous filtration	K R^2^	6.08 0.8564	11.5 0.7836	41.4 0.7034	17.3 0.9294	5.82 0.8874	11.1 0.8294	40.5 0.672	16.6 0.9426	10.2 0.6738	13.9 0.6678	50.5 0.7328	32.0 0.8226
Participation equation		TMP = 4.34TMP_b_ − 0.15TMP_i_ + 2.87TMPs − 1.99TMP_gl_				TMP = 6.91TMP_b_ − 4.62TMP_i_ + 6.24TMPs + 1.34TMP_gl_				TMP = 1.87TMP_b_ − 7.25TMP_i_ + 6.34TMPs + 5.39TMP_gl_			
*p*-value		1.2 × 10^−18^	4.17 × 10^−20^	1.85 × 10^−17^	1.2 × 10^−10^	6.7 × 10^−10^	8.9 × 10^−21^	1.4 × 10^−18^	1.5 × 10^−10^	0.037	9.6 × 10^−7^	1.9 × 10^−6^	5.5 × 10^−12^
Cycle 1	K R^2^	3.52 0.9768	6.99 0.9346	35.6 0.9413	7.28 0.9264	2.77 0.8637	5.35 0.7768	27.6 0.7843	5.5 0.7689	15.8 0.9785	11.3 0.9782	39.4 0.9828	29.6 0.9537
Participation equation		TMP = 0.32TMP_b_ +0.84TMP_i_ − 0.22TMPs + 0.07TMP_gl_				TMP = −0.31TMP_b_ + 2.46TMP_i_ − 0.39TMPs − 0.28TMP_gl_				TMP = 0.81TMP_b_ − 2.76TMP_i_ + 3.51TMPs + 1.36TMP_gl_			
*p*-value		0.92	0.74	0.71	0.51	0.96	0.85	0.86	0.93	0.026	0.034	0.046	0.016
Cycle 2	K R^2^	4.94 0.9583	8.17 0.9871	38.2 0.9748	10.7 0.9943	4.76 0.9775	9.71 0.9267	35.6 0.9927	10.3 0.9185	19.8 0.9775	12.8 0.9832	44.6 0.9267	30.5 0.9915
Participation equation		TMP = 1.02TMP_b_ − 0.23TMP_i_ + 0.09TMPs + 0.02TMP_gl_				TMP = 1.56TMP_b_ − 0.15TMP_i_ + 0.19TMPs + 0.24TMP_gl_				TMP = 0.91TMP_b_ − 3.42TMP_i_ + 4.24TMPs + 2.21TMP_gl_			
*p*-value		0.03	0.27	0.24	0.04	0.01	0.029	0.036	0.02	0.014	0.056	0.017	0.14
Cycle 3	K R^2^	5.83 0.7156	10.9 0.6348	39.1 0.8735	15.37 0.8687	5.28 0.8483	9.86 0.9997	42.87 0.7778	14.6 0.7612	21.68 0.4863	13.4 0.6489	48.04 0.7164	31.6 0.8076
Participation equation		TMP = 1.29TMP_b_ − 0.62TMP_i_ + 0.11TMPs − 0.04TMP_gl_				TMP = 2.91TMP_b_ − 1.69TMP_i_ + 3.14TMPs + 0.64TMP_gl_				TMP = 1.27TMP_b_ − 5.61TMP_i_ + 6.61TMPs + 2.91TMP_gl_			
*p*-value		9 × 10^−4^	0.86	0.96	4.9 × 10^−5^	0.018	0.034	0.019	1.9 × 10^−3^	4.7 × 10^−6^	0.02	0.018	2.6 × 10^−13^
***120 lmh***													
1 h continuous filtration	K R^2^	4.58 0.8393	6.11 0.78323	23.63 0.5971	8.303 0.9039	4.21 0.8717	5.67 0.8307	22.41 0.6939	7.52 0.9175	7.62 0.6202	9.23 0.6228	30.41 0.6823	15.35 0.7638
Participation equation		TMP = 4.55TMP_b_ − 6.62TMP_i_ + 2.52TMPs + 4.64TMP_gl_				TMP = 4.31TMP_b_ − 4.75TMP_i_ + 7.61TMPs + 4.57TMP_gl_				TMP = −1.80TMP_b_ − 7.35TMP_i_ + 7.24TMPs + 5.51TMP_gl_			
*p*-value		0.002	3.2 × 10^−3^	1.6 × 10^−4^	1.3 × 10^−4^	0.12	2.4 × 10^−3^	1.4 × 10^−4^	2.7 × 10^−3^	2.63 × 10^−21^	2.51 × 10^−11^	8.76 × 10^−5^	1.09 × 10^−34^
Cycle 1	K R^2^	2.96 0.9672	4.28 0.9562	10.44 0.9331	4.12 0.9197	2.58 0.9061	3.72 0.8884	17.07 0.8494	3.53 0.8349	5.59 0.6309	7.11 0.8467	25.00 0.9966	5.16 0.9742
Participation equation		TMP = 2.91TMP_b_ − 1.12TMP_i_ + 1.77TMPs − 1.04TMP_gl_				TMP = 2.86TMP_b_ − 0.82TMP_i_ + 0.52TMPs − 1.24TMP_gl_				TMP = −0.86TMP_b_ − 0.05TMP_i_ +0.06TMPs + 0.26TMP_gl_			
*p*-value		0.14	0.24	0.25	0.12	0.13	0.25	0.26	0.09	1.24 × 10^−4^	0.63	0.45	1.45 × 10^−12^
Cycle 2	K R^2^	4.35 0.8720	5.66 0.9937	15.33 0.7593	6.76 0.9614	4.11 0.8906	5.54 0.9972	18.36 0.7299	6.62 0.9712	6.23 0.8475	8.32 0.8345	26.32 0.80667	9.75 0.9805
Participation equation		TMP = 3.52TMP_b_ − 2.91TMP_i_ + 1.93TMPs + 0.31TMP_gl_				TMP = 3.45TMP_b_ − 2.12TMP_i_ + 3.23TMPs + 0.28TMP_gl_				TMP = −1.24TMP_b_ − 1.22TMP_i_ + 2.11TMPs + 1.35TMP_gl_			
*p*-value		0.017	0.041	0.023	7.6 × 10^−6^	0.026	0.047	0.012	2.9 × 10^−4^	3.37 × 10^−8^	0.019	0.28	2.87 × 10^−15^
Cycle 3	K R^2^	4.68 0.8978	6.74 0.9068	27.64 0.8152	8.376 0.9278	4.433 0.9378	6.43 0.9424	23.89 0.8703	7.65 0.8651	7.14 0.9271	8.33 0.9190	22.64 0.9541	12.03 0.5126
Participation equation		TMP = 4.09TMP_b_ − 3.12TMP_i_ + 2.14TMPs + 2.14TMP_gl_				TMP = 4.21TMP_b_ − 2.78TMP_i_ + 4.11TMPs + 0.19TMP_gl_				TMP = −1.29TMP_b_ − 2.71TMP_i_ + 3.64TMPs + 3.14TMP_gl_			
*p*-value		5.5 × 10^−11^	4.7 × 10^−12^	1.6 × 10^−14^	2.3 × 10^−9^	5.5 × 10^−4^	0.16	0.011	4.7 × 10^−7^	2.41 × 10^−6^	0.025	0.014	1.12 × 10^−14^
***160 lmh***													
1 h continuous filtration	K R^2^	3.97 0.6833	6.89 0.6032	18.64 0.6162	5.71 0.9031	8.41 0.8031	5.83 0.7546	17.40 0.5264	4.86 0.9446	8.37 0.6683	5.32 0.5746	17.31 0.5264	5.06 0.8970
Participation equation		TMP = 6.73TMP_b_ + 0.01TMP_i_ + 1.23TMPs + 5.93TMP_gl_				TMP = 3.14TMP_b_ − 5.67TMP_i_ + 7.92TMPs + 4.82TMP_gl_				TMP = −4.61TMP_b_ − 8.12TMP_i_ + 7.94TMPs + 6.14TMP_gl_			
*p*-value		4.69 × 10^−28^	2.59 × 10^−22^	2.48 × 10^−15^	3.64 × 10^−32^	3.88 × 10^−27^	3.32 × 10^−19^	4.56 × 10^−15^	6.99 × 10^−48^	8.11 × 10^−39^	3.98 × 10^−32^	2.63 × 10^−20^	1.32 × 10^−51^
Cycle 1	K R^2^	3.514 0.9992	3.52 0.9820	16.92 0.9962	7.32 0.5253	3.29 0.9905	3.28 0.9577	17.71 0.9821	7.07 0.6163	1.02 0.6635	3.19 0.9707	15.71 0.9671	3.48 0.9987
Participation equation		TMP = 2.61TMP_b_ − 1.43TMP_i_ + 0.31TMPs + 2.14TMP_gl_				TMP = 0.96TMP_b_ − 1.20TMP_i_ + 0.55TMPs + 0.23TMP_gl_				TMP = −0.11TMP_b_ − 0.08TMP_i_ + 0.01TMPs + 0.28TMP_gl_			
*p*-value		0.22	0.57	0.56	0.47	0.019	0.046	0.45	0.016	2.01 × 10^−6^	2.73 × 10^−4^	4.54 × 10^−5^	1.77 × 10^−33^
Cycle 2	K R^2^	5.86 0.6943	5.03 0.9586	27.82 0.8381	3.31 0.9123	5.81 0.7116	4.98 0.9632	27.57 0.8515	3.25 0.9020	4.97 0.8361	4.41 0.9746	21.77 0.9727	3.80 0.9997
Participation equation		TMP = 3.41TMP_b_ − 0.94TMP_i_ + 1.04TMPs + 3.16TMP_gl_				TMP = 1.57TMP_b_ − 1.34TMP_i_ + 1.74TMPs + 1.16TMP_gl_				TMP = −2.45TMP_b_ − 3.45TMP_i_ + 2.31TMPs + 1.34TMP_gl_			
*p*-value		3.57 × 10^−3^	0.065	0.055	5.64 × 10^−6^	1.35 × 10^−3^	0.049	0.041	8.73 × 10^−6^	3.75 × 10^−7^	0.021	0.029	2.42 × 10^−17^
Cycle 3	K R^2^	8.32 0.9321	6.27 0.9653	29.32 0.9657	3.32 0.9996	3.50 0.9906	4.15 0.9992	23.48 0.9774	3.78 0.9632	5.32 0.9789	4.26 0.9863	33.13 0.7032	3.92 0.9979
Participation equation		TMP = 3.96TMP_b_ − 0.04TMP_i_ + 1.96TMPs + 4.02TMP_gl_				TMP = 2.25TMP_b_ − 2.94TMP_i_ + 3.25TMPs + 1.98TMP_gl_				TMP = −4.12TMP_b_ − 5.94TMP_i_ + 4.04TMPs + 2.16TMP_gl_			
*p*-value		2.15 × 10^−4^	0.085	0.049		3.39 × 10^−4^	0.073	0.013	6.61 × 10^−9^	1.48 × 10^−7^	0.012	0.046	5.15 × 10^−15^
***200 lmh***													
1 h continuous filtration	K R^2^	3.05 0.9262	2.45 0.9355	10.97 0.8234	3.61 0.9834	2.53 0.9903	2.04 0.9804	9.851 0.9532	2.88 0.9932	5.31 0.7322	2.67 0.9028	11.23 0.8153	3.26 0.9676
Participation equation		TMP = 7.95TMP_b_ + 0.65TMP_i_ − 0.21TMPs + 7.83TMP_gl_				TMP = 2.09TMP_b_ − 6.87TMP_i_ + 8.34TMPs + 4.97TMP_gl_				TMP = −7.64TMP_b_ − 9.12TMP_i_ + 9.89TMPs + 8.64TMP_gl_			
*p*-value		8.53 × 10^−13^	1.28 × 10^−9^	0.014	9.95 × 10^−19^	1.93 × 10^−10^	7.89 × 10^−5^	7.4 × 10^−4^	2.6 × 10^−12^	6.81 × 10^−29^	1.75 × 10^−22^	3.52 × 10^−13^	2.08 × 10^−36^
Cycle 1	K R^2^	2.87 0.9832	2.55 0.9815	17.07 0.9512	2.70 0.9921	2.75 0.9692	2.43 0.9668	16.55 0.9832	2.57 0.9679	5.11 0.6685	4.49 0.6621	21.87 0.6238	3.56 0.9821
Participation equation		TMP = 0.49TMP_b_ − 0.03TMP_i_ + 0.08TMPs +0.16TMP_gl_				TMP = 0.27TMP_b_ − 0.07TMP_i_ + 0.02TMPs +0.18TMP_gl_				TMP = 0.09TMP_b_ − 0.04TMP_i_ + 3.98TMPs + 0.29TMP_gl_			
*p*-value		8.86 × 10^−4^	0.65	0.59	2.64 × 10^−5^	9.18 × 10^−4^	0.046	0.039	9.64 × 10^−7^	8.68 × 10^−7^	0.012	0.066	8.17 × 10^−14^
Cycle 2	K R^2^	3.06 0.9956	2.71 0.9945	13.53 0.9933	1.60 0.7273	3.03 0.9937	2.69 0.9927	13.41 0.9991	1.59 0.7158	4.34 0.9075	3.86 0.9008	19.20 0.8782	3.44 0.9946
Participation equation		TMP = 0.87TMP_b_ + 0.10TMP_i_ + 0.01TMPs +0.15TMP_gl_				TMP = 0.98TMP_b_ − 0.98TMP_i_ + 0.87TMPs +0.69TMP_gl_				TMP = −0.06TMP_b_ − 0.69TMP_i_ + 4.19TMPs + 1.56TMP_gl_			
*p*-value		1.5 × 10^−4^	0.023	0.087	5.8 × 10^−7^	4.33 × 10^−5^	0.073	0.063	2.16 × 10^−6^	1.75 × 10^−7^	0.86	0.023	1.6 × 10^−12^
Cycle 3	K R^2^	4.02 0.9367	3.58 0.9319	17.90 0.9157	3.08 0.9981	3.95 0.9492	3.52 0.9450	14.90 0.9987	3.01 0.9990	4.01 0.9591	3.33 0.9857	20.19 0.8121	3.17 0.9997
Participation equation		TMP = 1.81TMP_b_ + 1.24TMP_i_ + 0.84TMPs + 0.21TMP_gl_				TMP = 2.34TMP_b_ − 2.14TMP_i_ + 1.59TMPs + 1.63TMP_gl_				TMP = −1.93TMP_b_ − 1.31TMP_i_ + 6.13TMPs +2.31TMP_gl_			
*p*-value		5.72 × 10^−6^	0.04	0.03	3.04 × 10^−13^	9.12 × 10^−7^	0.85	0.61	9.28 × 10^−10^	9.57 × 10^−8^	0.93	0.14	1.72 × 10^−12^

## Data Availability

The authors agree to make data available on request.

## Supplementary Materials

The following supporting information can be downloaded at: https://www.mdpi.com/article/10.3390/membranes13110853/s1. Figure S1: Schematic representation of Hermia’s fouling model mechanisms; Figure S2: Lab scale membrane system; Figure S3: Raise in TMP of membrane under testing for raise in flux with 20 LMH from 40–200 LMH for DI water. Figure S4: Represents the fitted fouling curve for the fouling solution at 40 lmh for 1 h filtration; a: S1 solution; b: S2 solution; c: S3 solution (subscript 1,2, 3 & 4 represents complete, intermediate, standard and cake layer blocking). Figure S5: Represents the fitted fouling curve for the fouling solution at 40 lmh for cycle 1 filtration; a: S1 solution; b: S2 solution; c: S3 solution (subscript 1,2, 3 & 4 represents complete, intermediate, standard and cake layer blocking). Figure S6: Represents the fitted fouling curve for the fouling solution at 40 lmh for cycle 2 filtration; a: S1 solution; b: S2 solution; c: S3 solution (subscript 1,2, 3 & 4 represents complete, intermediate, standard and cake layer blocking). Figure S7: Represents the fitted fouling curve for the fouling solution at 40 lmh for cycle 3 filtration; a: S1 solution; b: S2 solution; c: S3 solution (subscript 1,2, 3 & 4 represents complete, intermediate, standard and cake layer blocking). Figure S8: Represents the fitted fouling curve for the fouling solution at 80 lmh for 1 h filtration; a: S1 solution; b: S2 solution; c: S3 solution (subscript 1, 2, 3 & 4 represents complete, intermediate, standard and cake layer blocking). Figure S9: Represents the fitted fouling curve for the fouling solution at 80 lmh for cycle 1 filtration; a: S1 solution; b: S2 solution; c: S3 solution (subscript 1, 2, 3 & 4 represents complete, intermediate, standard and cake layer blocking). Figure S10: Represents the fitted fouling curve for the fouling solution at 80 lmh for cycle 2 filtration; a: S1 solution; b: S2 solution; c: S3 solution (subscript 1, 2, 3 & 4 represents complete, intermediate, standard and cake layer blocking). Figure S11: Represents the fitted fouling curve for the fouling solution at 80 lmh for cycle 3 filtration; a: S1 solution; b: S2 solution; c: S3 solution (subscript 1, 2, 3 & 4 represents complete, intermediate, standard and cake layer blocking). Figure S12: Represents the fitted fouling curve for the fouling solution at 120 lmh for 1 h filtration; a: S1 solution; b: S2 solution; c: S3 solution (subscript 1, 2, 3 & 4 represents complete, intermediate, standard and cake layer blocking). Figure S13: Represents the fitted fouling curve for the fouling solution at 120 lmh for cycle 1 filtration; a: S1 solution; b: S2 solution; c: S3 solution (subscript 1, 2, 3 & 4 represents complete, intermediate, standard and cake layer blocking). Figure S14: Represents the fitted fouling curve for the fouling solution at 120 lmh for cycle 2 filtration; a: S1 solution; b: S2 solution; c: S3 solution (subscript 1, 2, 3 & 4 represents complete, intermediate, standard and cake layer blocking). Figure S15: Represents the fitted fouling curve for the fouling solution at 120 lmh for cycle 3 filtration; a: S1 solution; b: S2 solution; c: S3 solution (subscript 1, 2, 3 & 4 represents complete, intermediate, standard and cake layer blocking). Figure S16: Represents the fitted fouling curve for the fouling solution at 160 lmh for 1 h filtration; a: S1 solution; b: S2 solution; c: S3 solution (subscript 1, 2, 3 & 4 represents complete, intermediate, standard and cake layer blocking). Figure S17: Represents the fitted fouling curve for the fouling solution at 160 lmh for Cycle 1 filtration; a: S1 solution; b: S2 solution; c: S3 solution (subscript 1, 2, 3 & 4 represents complete, intermediate, standard and cake layer blocking). Figure S18: Represents the fitted fouling curve for the fouling solution at 160lmh for Cycle 2 filtration; a: S1 solution; b: S2 solution; c: S3 solution (subscript 1, 2, 3 & 4 represents complete, intermediate, standard and cake layer blocking). Figure S19: Represents the fitted fouling curve for the fouling solution at 160 lmh for Cycle 3 filtration; a: S1 solution; b: S2 solution; c: S3 solution (subscript 1, 2, 3 & 4 represents complete, intermediate, standard and cake layer blocking). Figure S20: Represents the fitted fouling curve for the fouling solution at 200 lmh for 1 h filtration; a: S1 solution; b: S2 solution; c: S3 solution (subscript 1, 2, 3 & 4 represents complete, intermediate, standard and cake layer blocking). Figure S21: Represents the fitted fouling curve for the fouling solution at 200 lmh for Cycle 1 filtration; a: S1 solution; b: S2 solution; c: S3 solution (subscript 1, 2, 3 & 4 represents complete, intermediate, standard and cake layer blocking). Figure S22: Represents the fitted fouling curve for the fouling solution at 200lmh for Cycle 2 filtration; a: S1 solution; b: S2 solution; c: S3 solution (subscript 1, 2, 3 & 4 represents complete, intermediate, standard and cake layer blocking). Figure S23: Represents the fitted fouling curve for the fouling solution at 200 lmh for Cycle 3 filtration; a: S1 solution; b: S2 solution; c: S3 solution (subscript 1, 2, 3 & 4 represents complete, intermediate, standard and cake layer blocking). Table S1: PVDF membrane characteristics and operational conditions. Table S2: Feed solution characteristics. Table S3: Summary of the foulants associated with membrane processes used for surface water treatment. References [41,42,43,44,45,46] are cited in the supplementary materials.
