# Supplementary material for: Evaluation of Organic and Inorganic Foulant Interaction Using Modified Fouling Models in Constant Flux Dead-End Operation with Microfiltration Membranes
Source: Membranes (Basel). 2023 Oct 25;13(11):853. doi: 10.3390/membranes13110853 (PMC10673472; doi:10.3390/membranes13110853)
Supplement: Supplementary file 1 [file membranes-13-00853-s001.zip › membranes-2640052-supplementary.pdf]

Evaluation of organic and inorganic foulant interaction using modified fouling models in constant flux dead end operation with microfiltration membranes

Muhammad Qasim[1], Ali Akbar[2], Imtiaz Afzal Khan[3], Mumtaz Ali[4], Eui-Jong Lee[5] and Kang Hoon Lee[6*]

[1]Department of Civil Engineering, The University of Lahore, Lahore Campus, 1-km defense road, Lahore, Pakistan; Muhammad.qasim@ce.uol.edu.pk

[2]Department of Mechanical Engineering, University of Engineering and Technology Lahore (Rachna Campus), Pakistan; ali.akbar@uet.edu.pk

[3]Interdisciplinary Research Center for Membranes and Water Security, King Fahd University of Petroleum & Minerals (KFUPM), Dhahran, 31261 Saudi Arabia; imtiaz.khan@kfupm.edu.sa

[4]Department of Textile Engineering, National Textile University, Faisalabad 37610, Pakistan; mumtaz@ntu.edu.pk

[5]Department of Environmental Engineering, Daegu University, 201 Daegudae-ro, Jillyang,Gyeongsan-si 38453, Republic of Korea; lujong@daegu.ac.kr

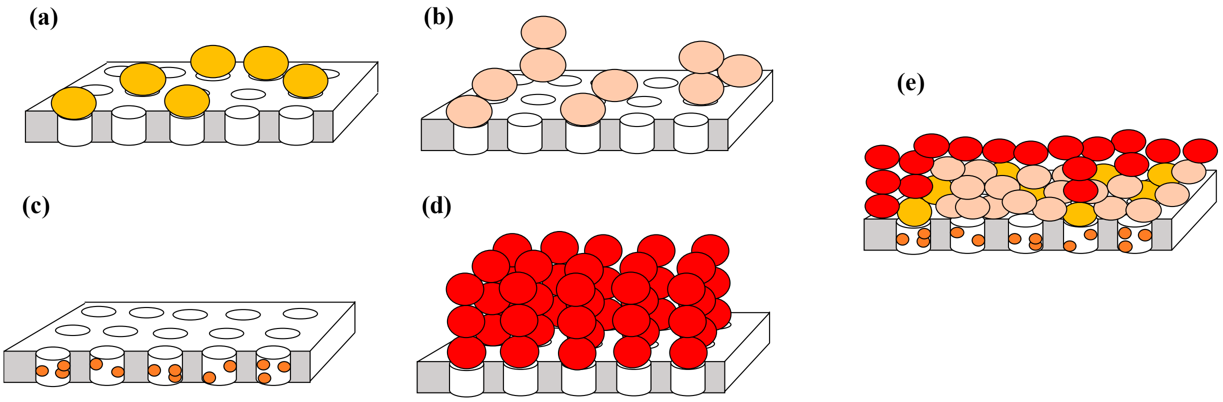

Fig S1. Schematic representation of Hermia's fouling model mechanisms: (a) complete fouling, (b) intermediate fouling, (c) standard fouling, (d) cake layer fouling and (e) combined fouling model distribution during the process

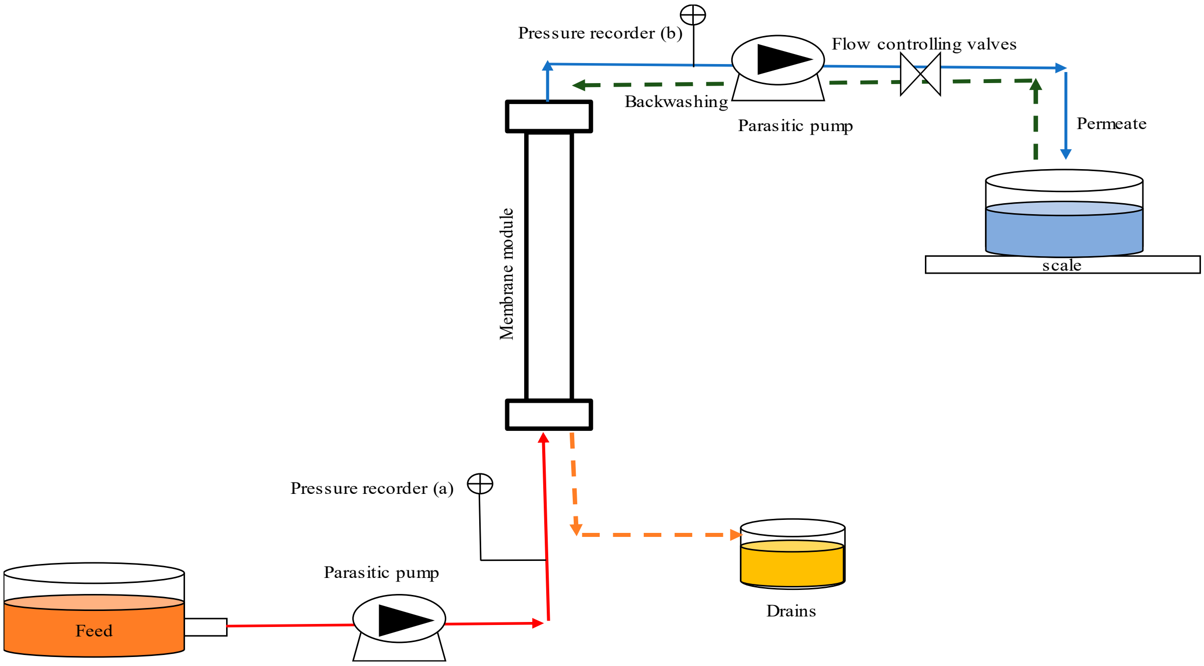

Fig S2: Lab scale membrane system

Table S1: PVDF membrane characteristics and operational conditions

Parameters	Detail
Membrane pore size	0.1μm
Outer diameter	1.4mm
Inner diameter	0.6mm
Membrane area	$0.1884 m^2$
pH	8.5-9.5
Operating temperature	18 ± 3^0C
Flux range	40-200 lmh

Table S2: Feed solution characteristics

Sample name	Feed solution	Particle size (μm)
S1	HA + Ca^{+2}	0.213 ± 0.11
S2	Al + Mn + Fe + Ca^{+2} +Kaolin	0.156 ± 0.08
S3	HA+ Al + Mn + Fe + Ca^{+2} +Kaolin	0.268 ± 0.13

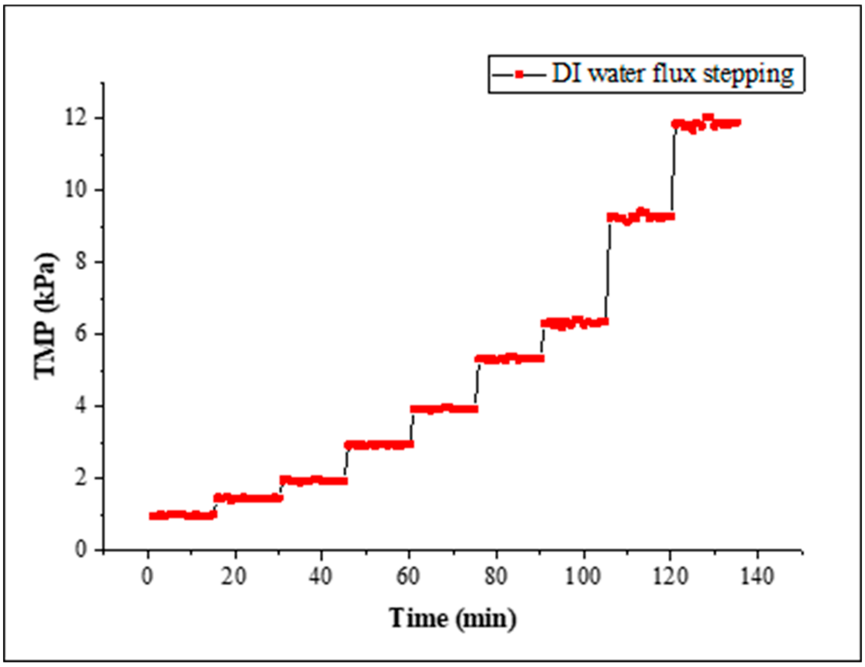

Fig. S3 : Raise in TMP of membrane under testing for raise in flux with 20 LMH from 40 – 200 LMH for DI water.

1. Fitting curves for modified fouling model for the 40lmh

1.1.1hr filtration

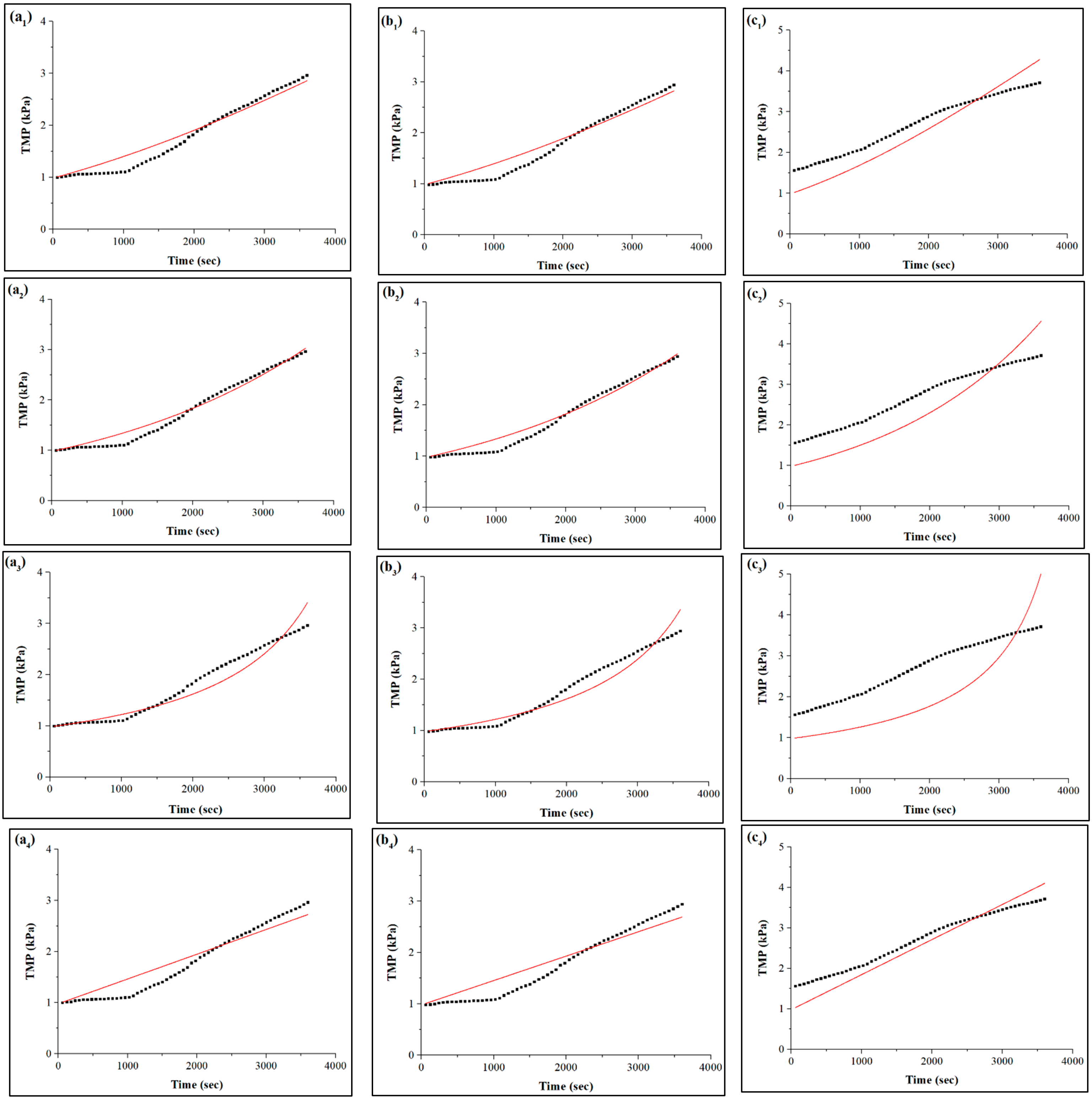

Fig. S4: Represents the fitted fouling curve for the fouling solution at 40lmh for 1hr filtration; a: S1 solution; b: S2 solution; c: S3 solution (subscript 1,2,3 & 4 represents complete, intermediate, standard and cake layer blocking)

1.2.Cycle 1 filtration (40lmh)

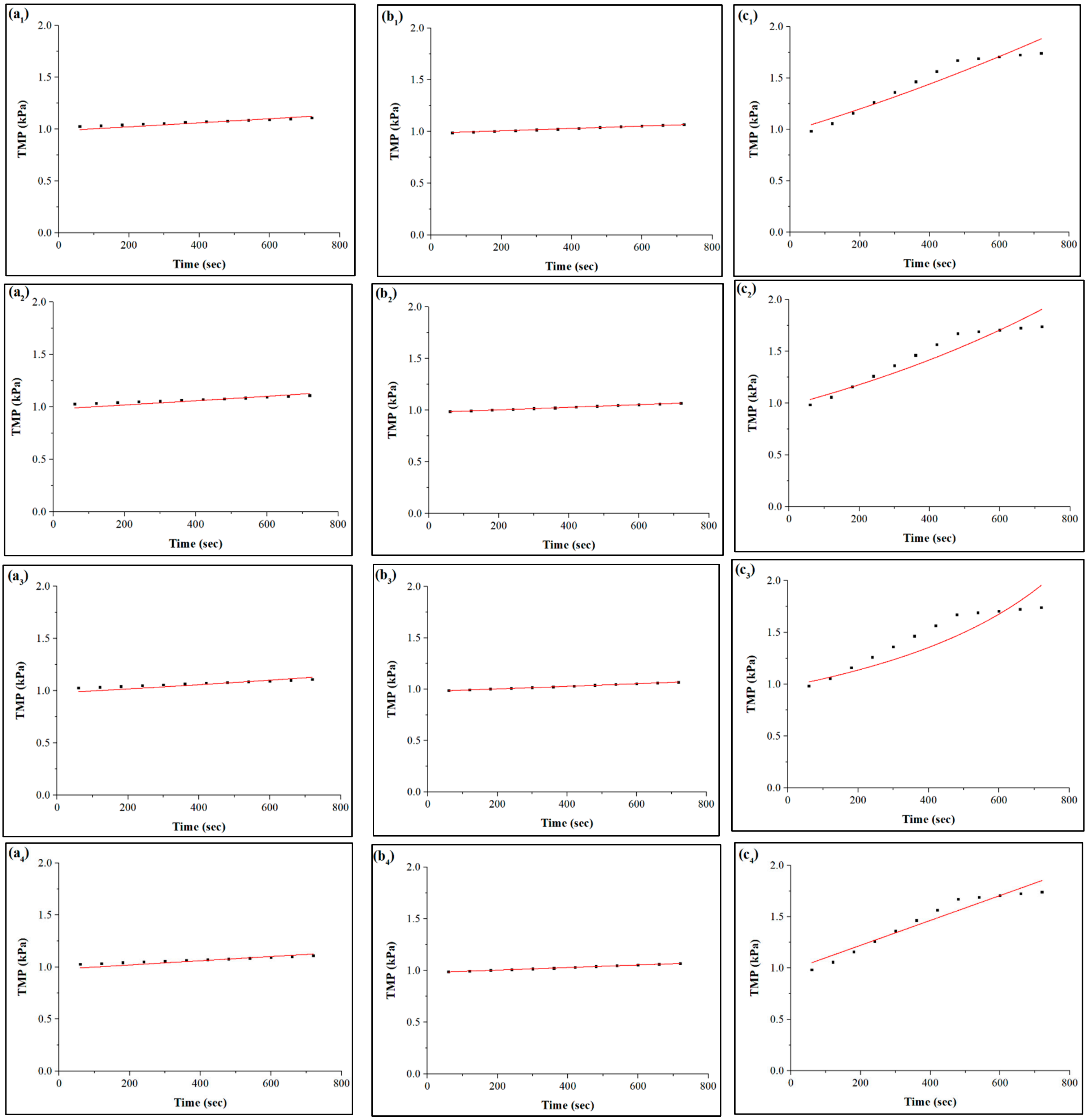

Fig. S5: Represents the fitted fouling curve for the fouling solution at 40lmh for cycle 1 filtration; a: S1 solution; b: S2 solution; c: S3 solution (subscript 1,2,3 & 4 represents complete, intermediate, standard and cake layer blocking)

1.3.Cycle 2 filtration (40 lmh)

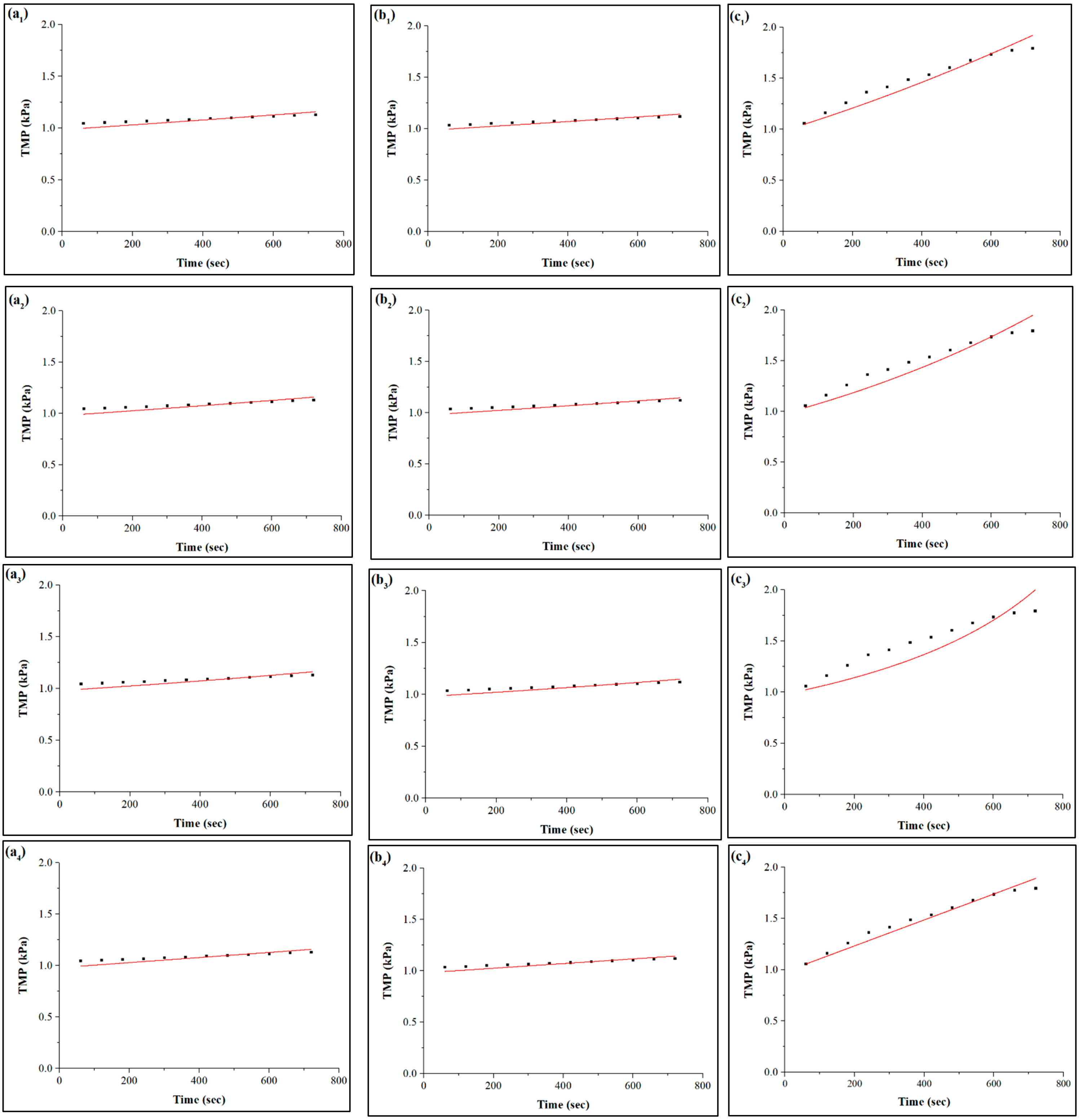

Fig. S6: Represents the fitted fouling curve for the fouling solution at 40lmh for cycle 2 filtration; a: S1 solution; b: S2 solution; c: S3 solution (subscript 1,2,3 & 4 represents complete, intermediate, standard and cake layer blocking)

1.4.Cycle 3 filtration (40lmh)

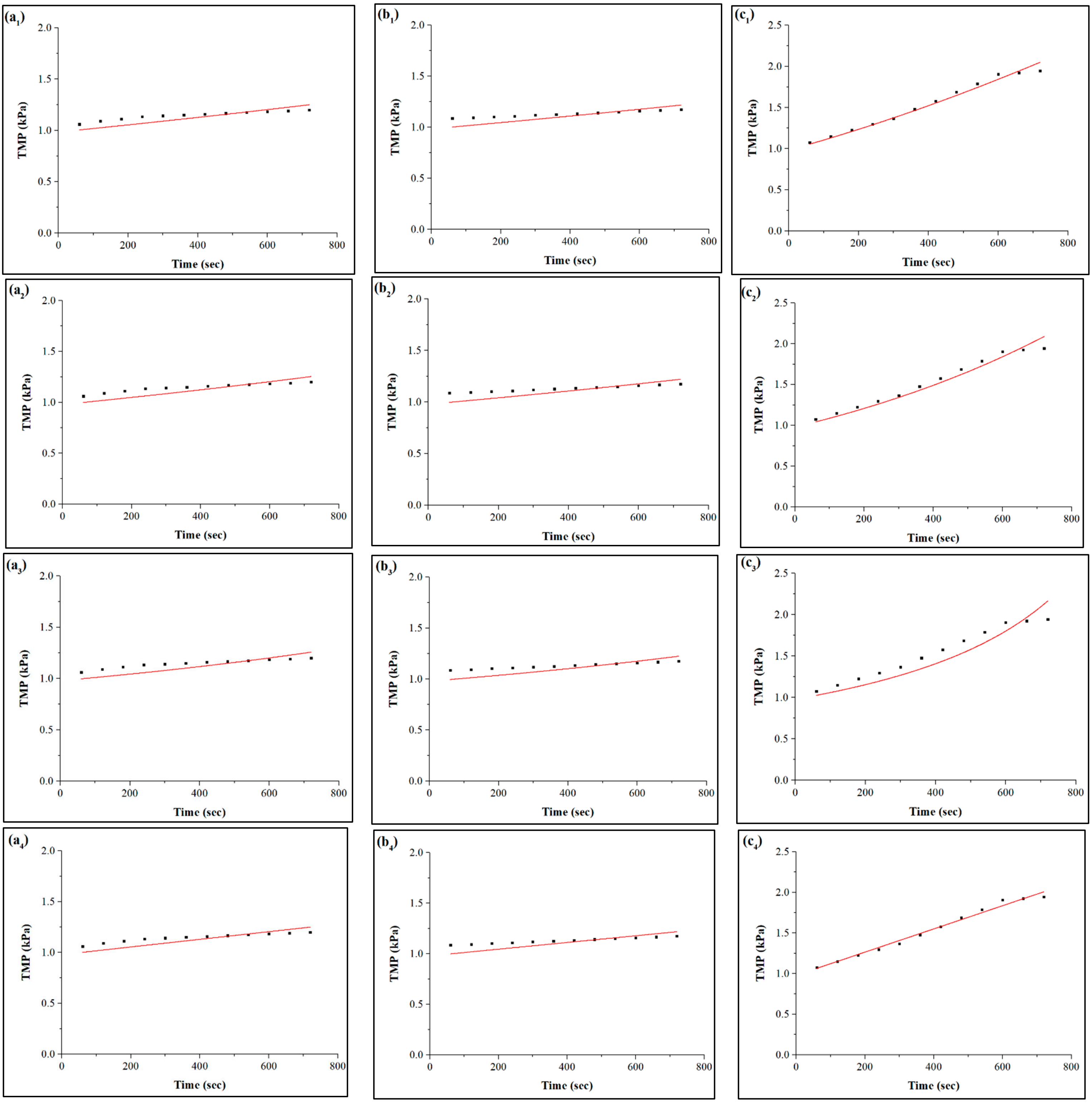

Fig. S7: Represents the fitted fouling curve for the fouling solution at 40lmh for cycle 3 filtration; a: S1 solution; b: S2 solution; c: S3 solution (subscript 1,2,3 & 4 represents complete, intermediate, standard and cake layer blocking)

2. Fitting curves for modified fouling model for the 80lmh

2.1. 1hr filtration

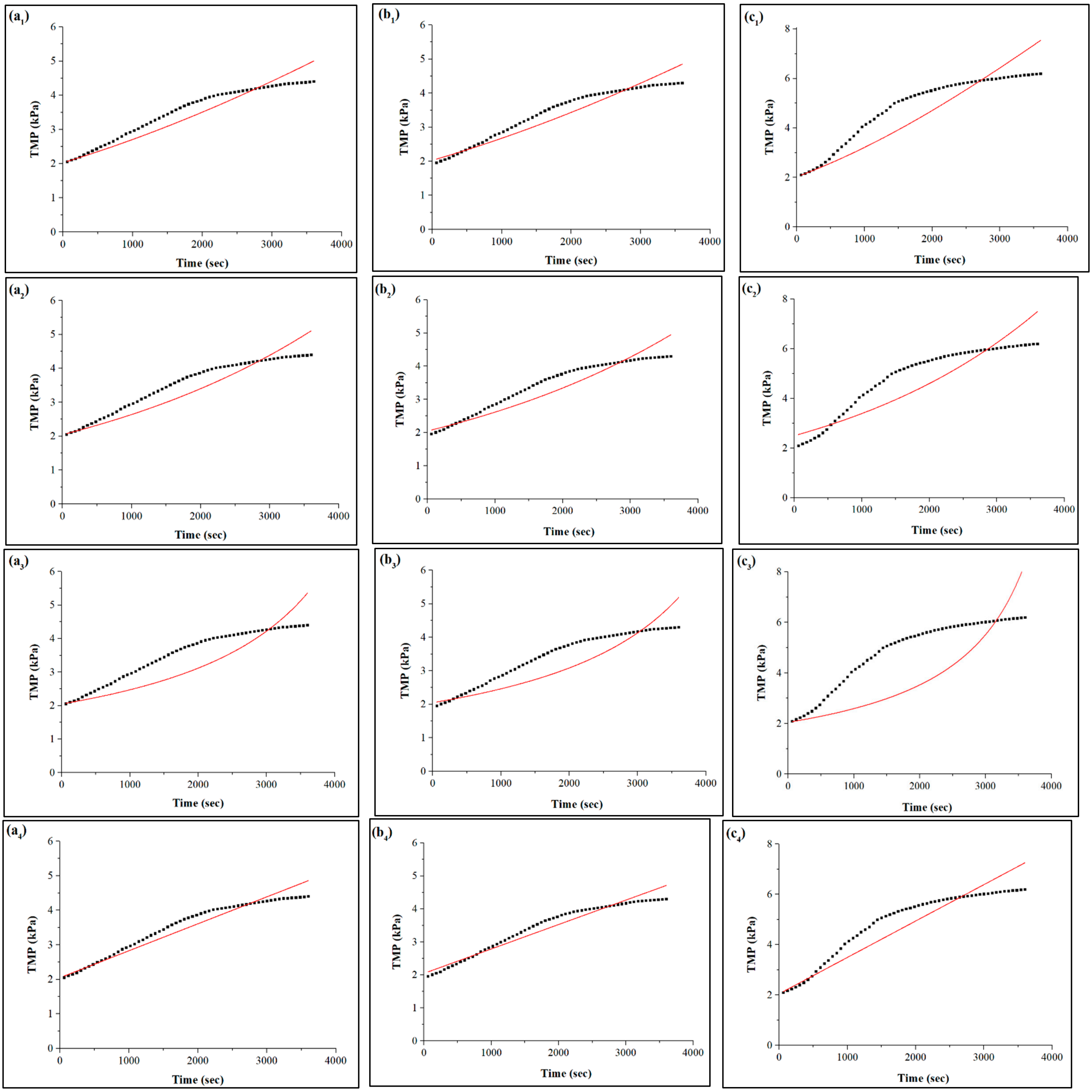

Fig. S8: Represents the fitted fouling curve for the fouling solution at 80lmh for 1hr filtration; a: S1 solution; b: S2 solution; c: S3 solution (subscript 1,2,3 & 4 represents complete, intermediate, standard and cake layer blocking)

2.2.Cycle 1filtration (80 lmh)

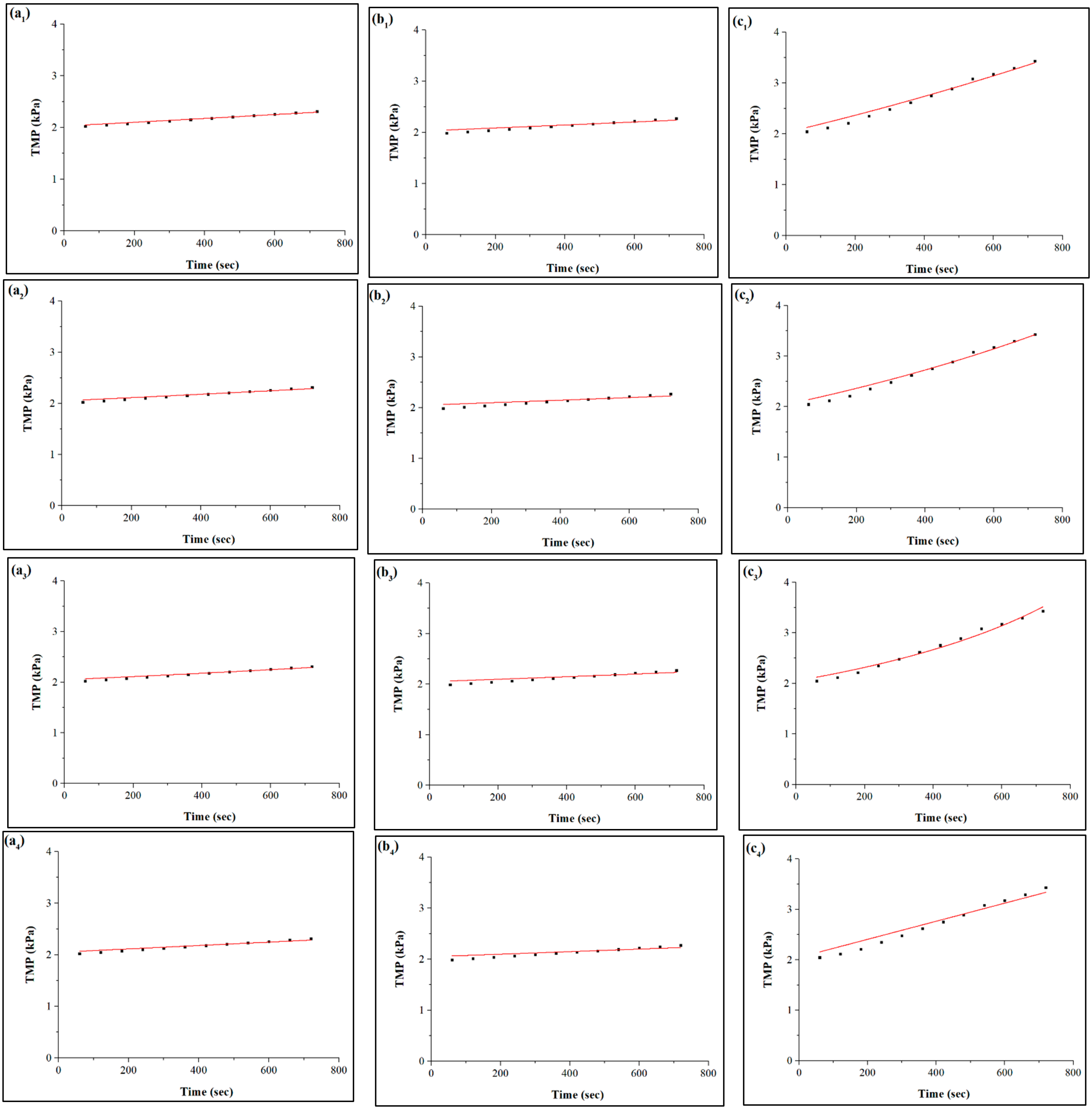

Fig. S9: Represents the fitted fouling curve for the fouling solution at 80lmh for cycle 1 filtration; a: S1 solution; b: S2 solution; c: S3 solution (subscript 1, 2,3 & 4 represents complete, intermediate, standard and cake layer blocking)

2.3. Cycle 2 filtration (80 lmh)

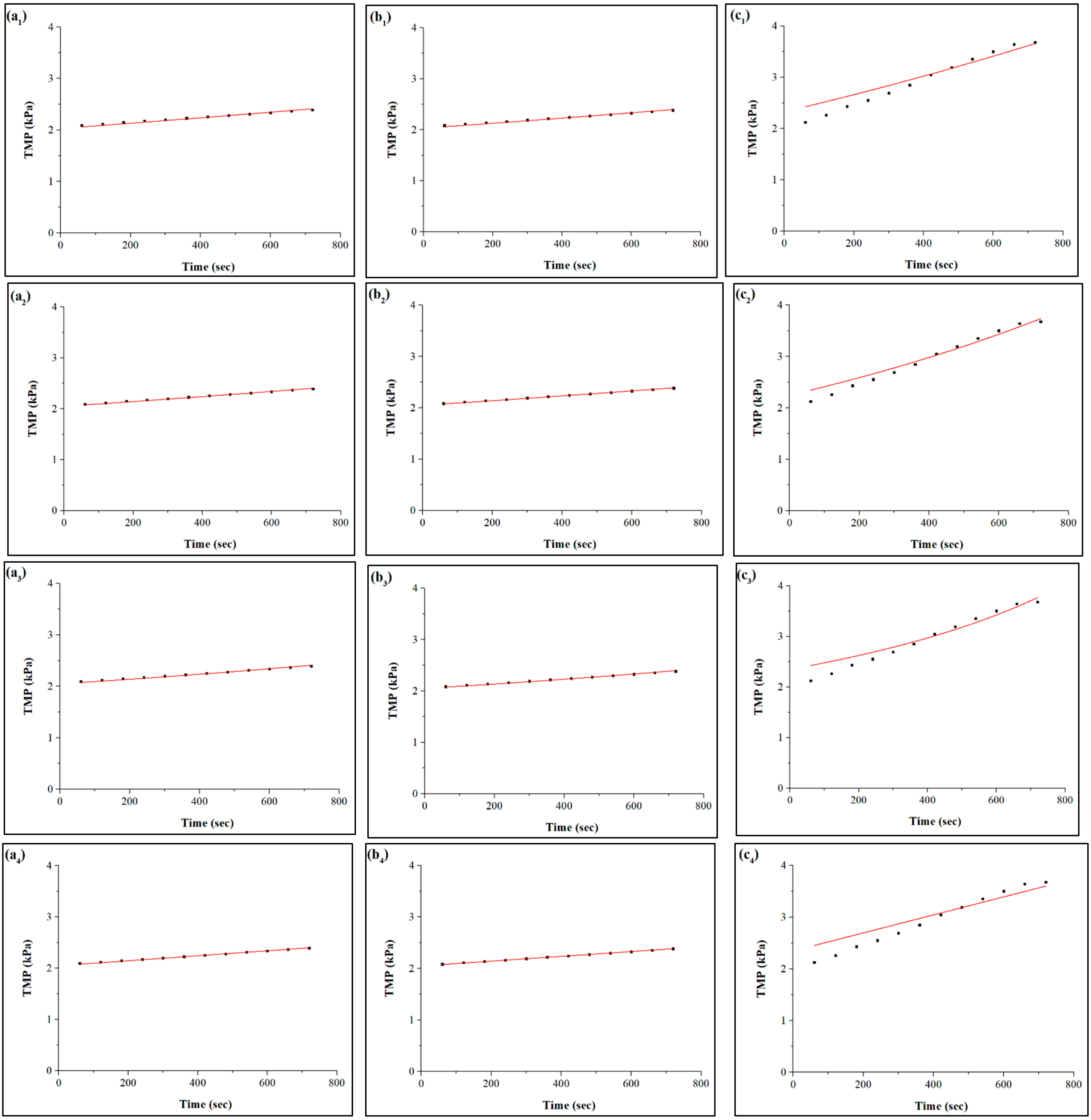

Fig. S10: Represents the fitted fouling curve for the fouling solution at 80lmh for cycle 2 filtration; a: S1 solution; b: S2 solution; c: S3 solution (subscript 1, 2,3 & 4 represents complete, intermediate, standard and cake layer blocking)

2.4. Cycle 3 filtration (80lmh)

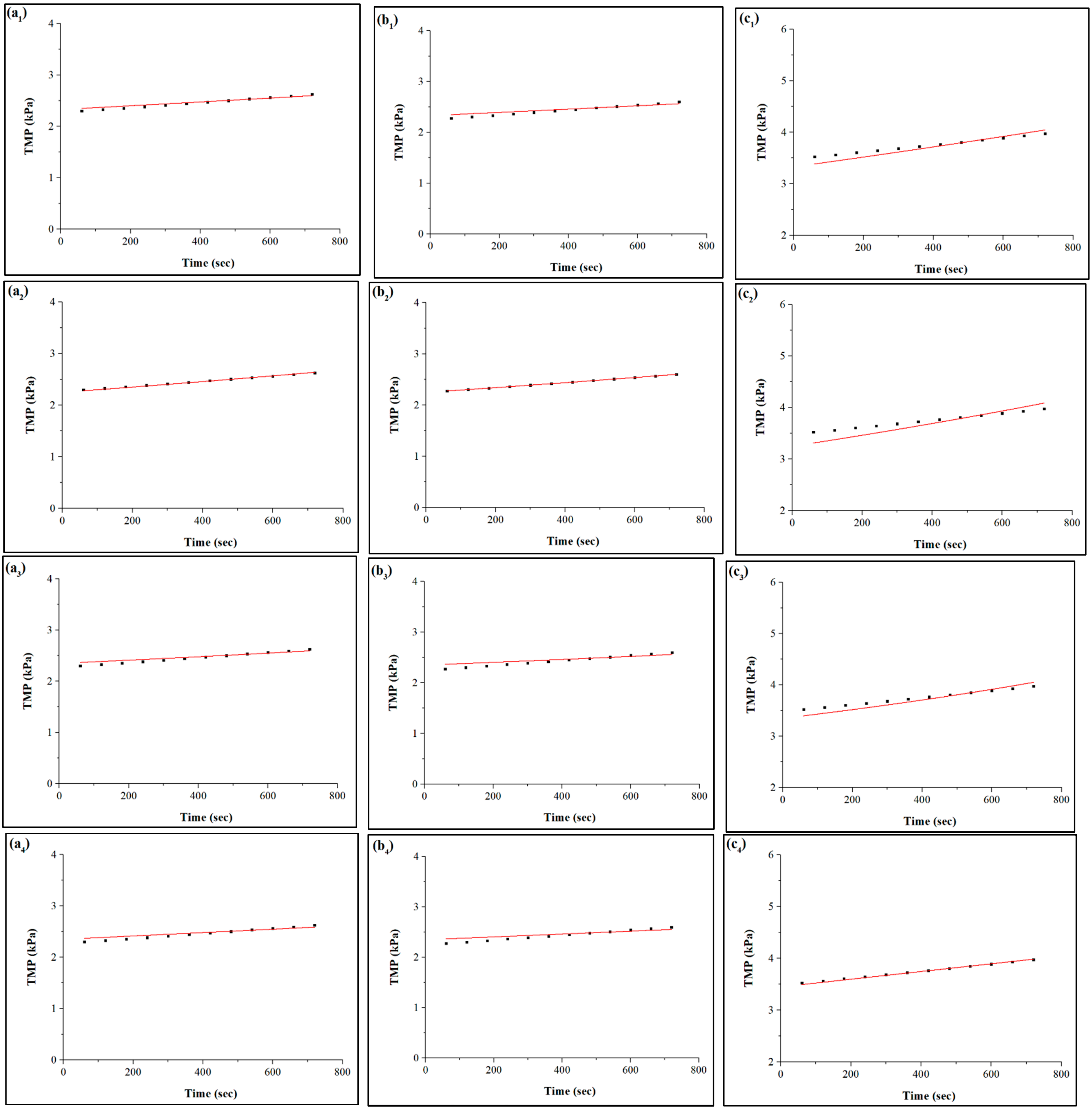

Fig. S11: Represents the fitted fouling curve for the fouling solution at 80lmh for cycle 3 filtration; a: S1 solution; b: S2 solution; c: S3 solution (subscript 1, 2,3 & 4 represents complete, intermediate, standard and cake layer blocking)

3. Fitting curves for modified fouling model for the 120lmh

3.1. 1hr filtration

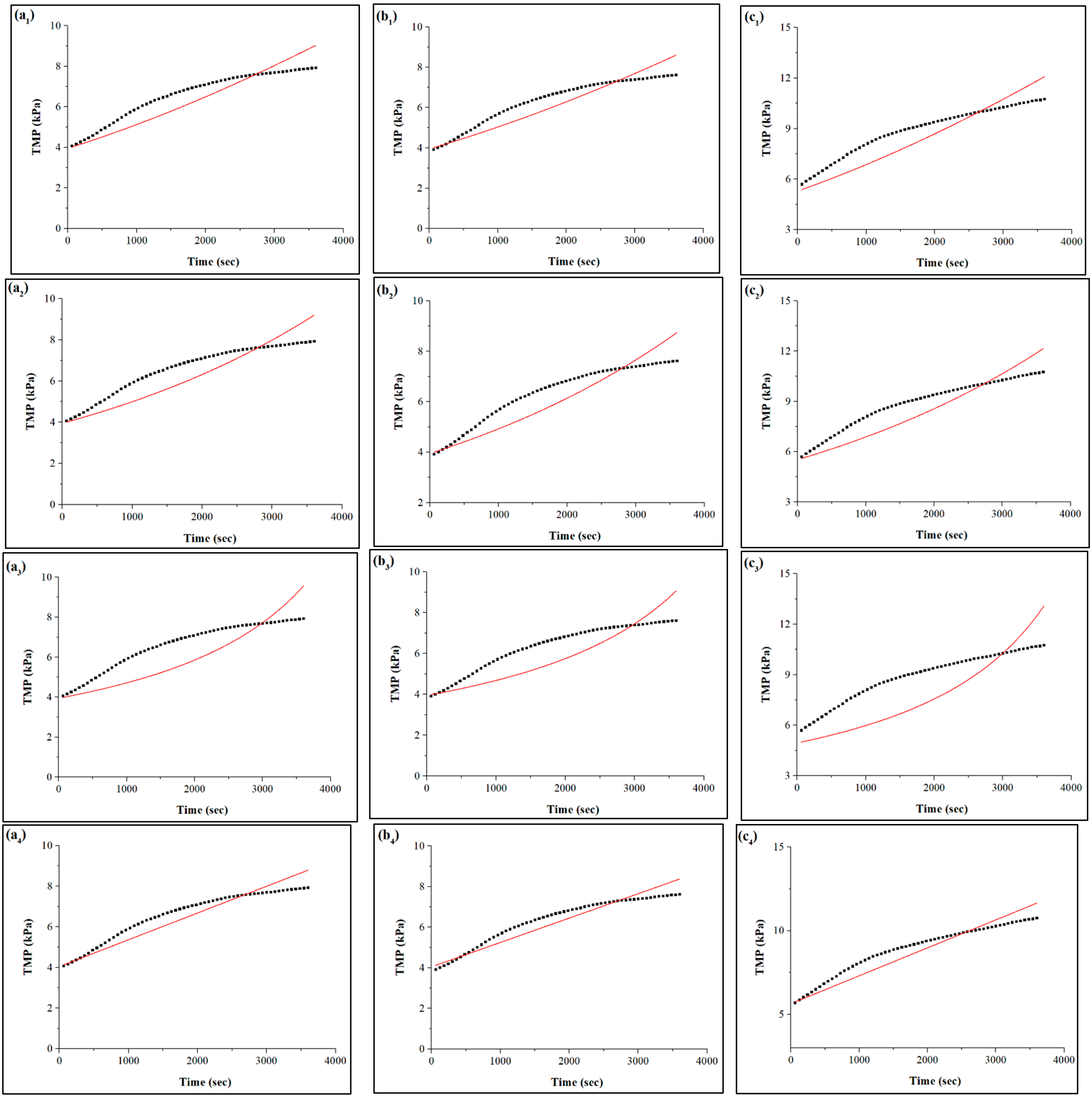

Fig. S12: Represents the fitted fouling curve for the fouling solution at 120lmh for 1hr filtration; a: S1 solution; b: S2 solution; c: S3 solution (subscript 1, 2,3 & 4 represents complete, intermediate, standard and cake layer blocking)

3.2. Cycle 1 filtration (120 lmh)

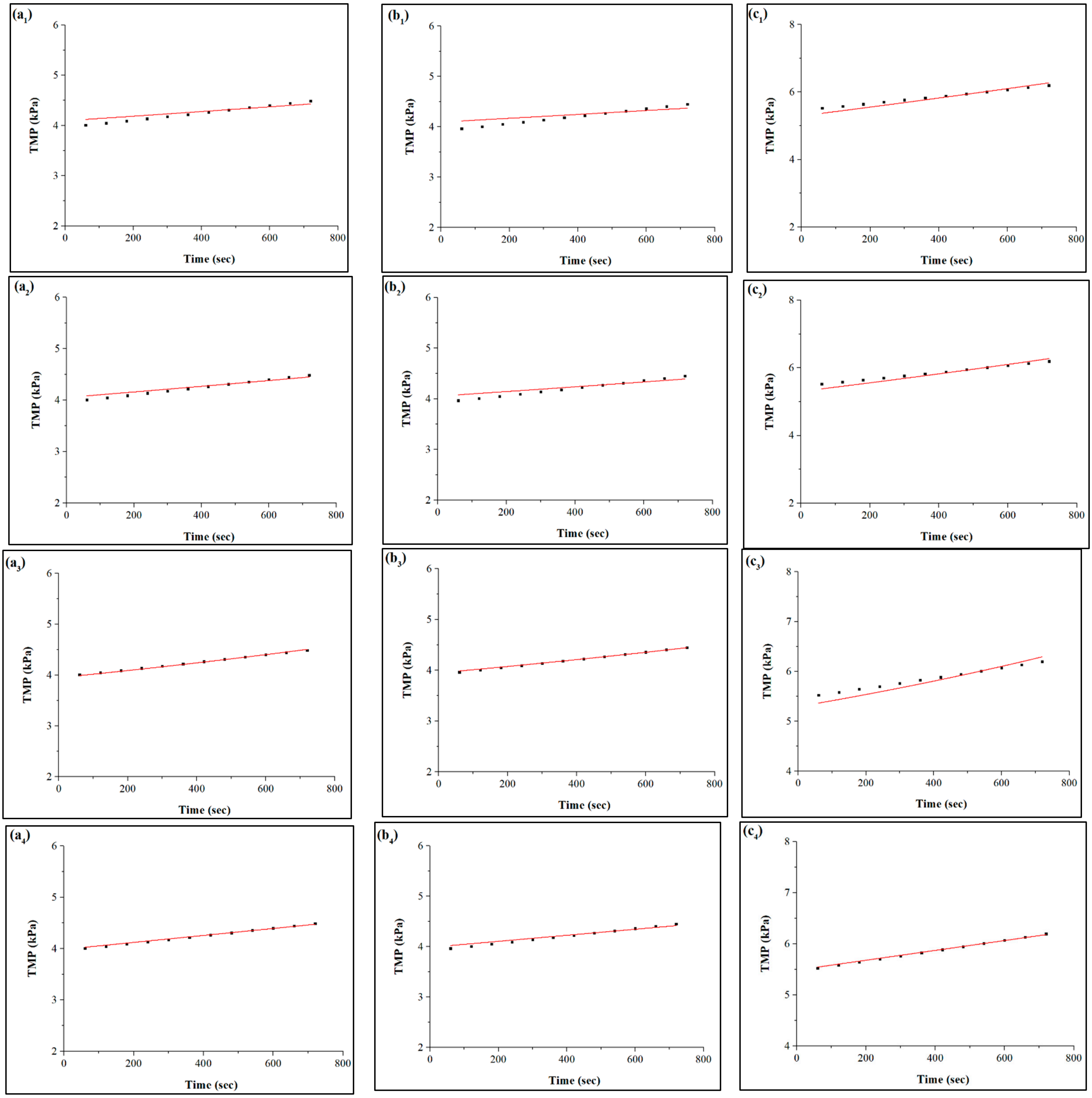

Fig. S13: Represents the fitted fouling curve for the fouling solution at 120lmh for cycle 1 filtration; a: S1 solution; b: S2 solution; c: S3 solution (subscript 1, 2,3 & 4 represents complete, intermediate, standard and cake layer blocking)

3.3. Cycle 2 filtration (120 lmh)

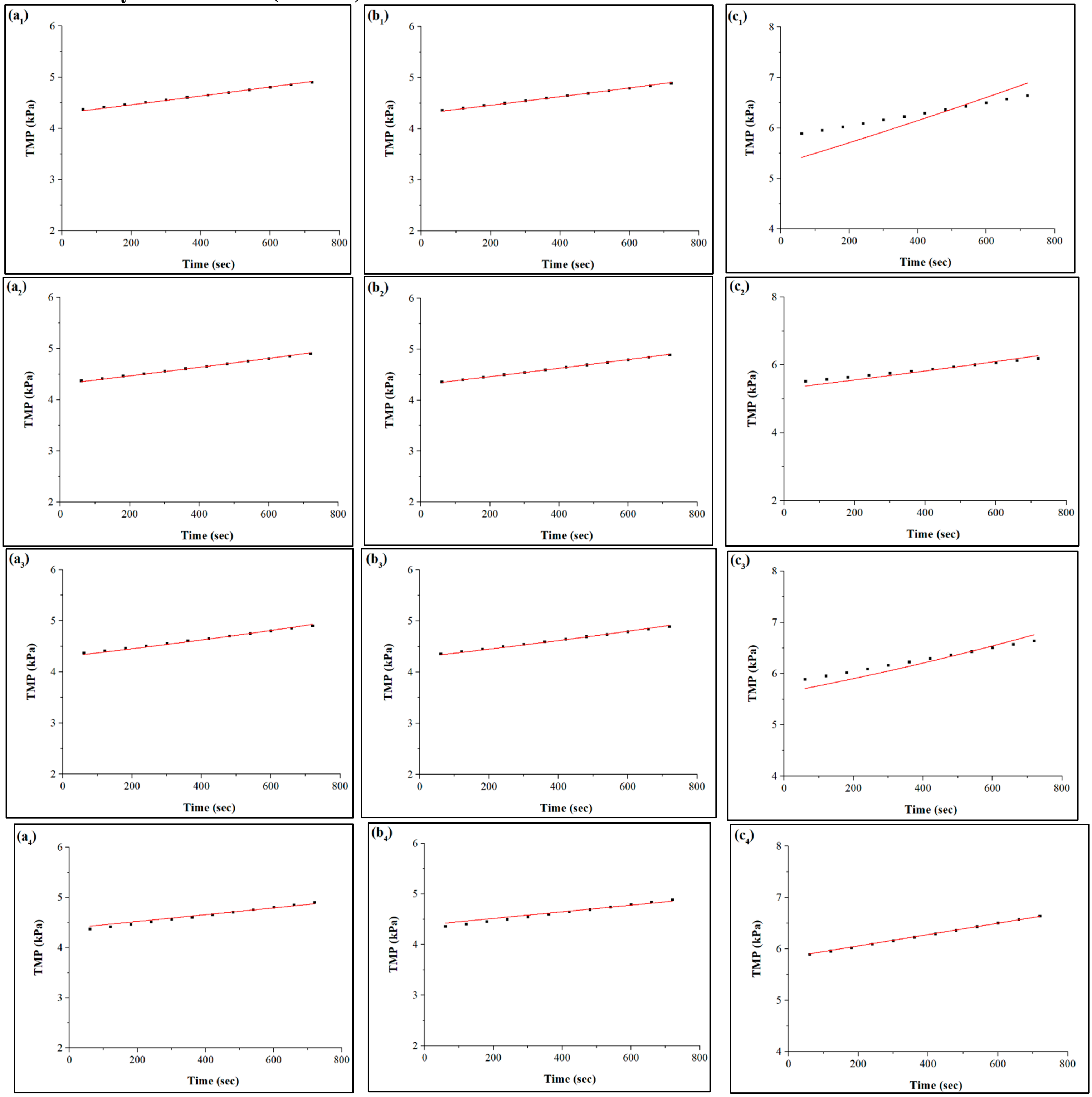

Fig. S14: Represents the fitted fouling curve for the fouling solution at 120lmh for cycle 2 filtration; a: S1 solution; b: S2 solution; c: S3 solution (subscript 1, 2,3 & 4 represents complete, intermediate, standard and cake layer blocking)

3.4.Cycle 3 filtration (120 lmh)

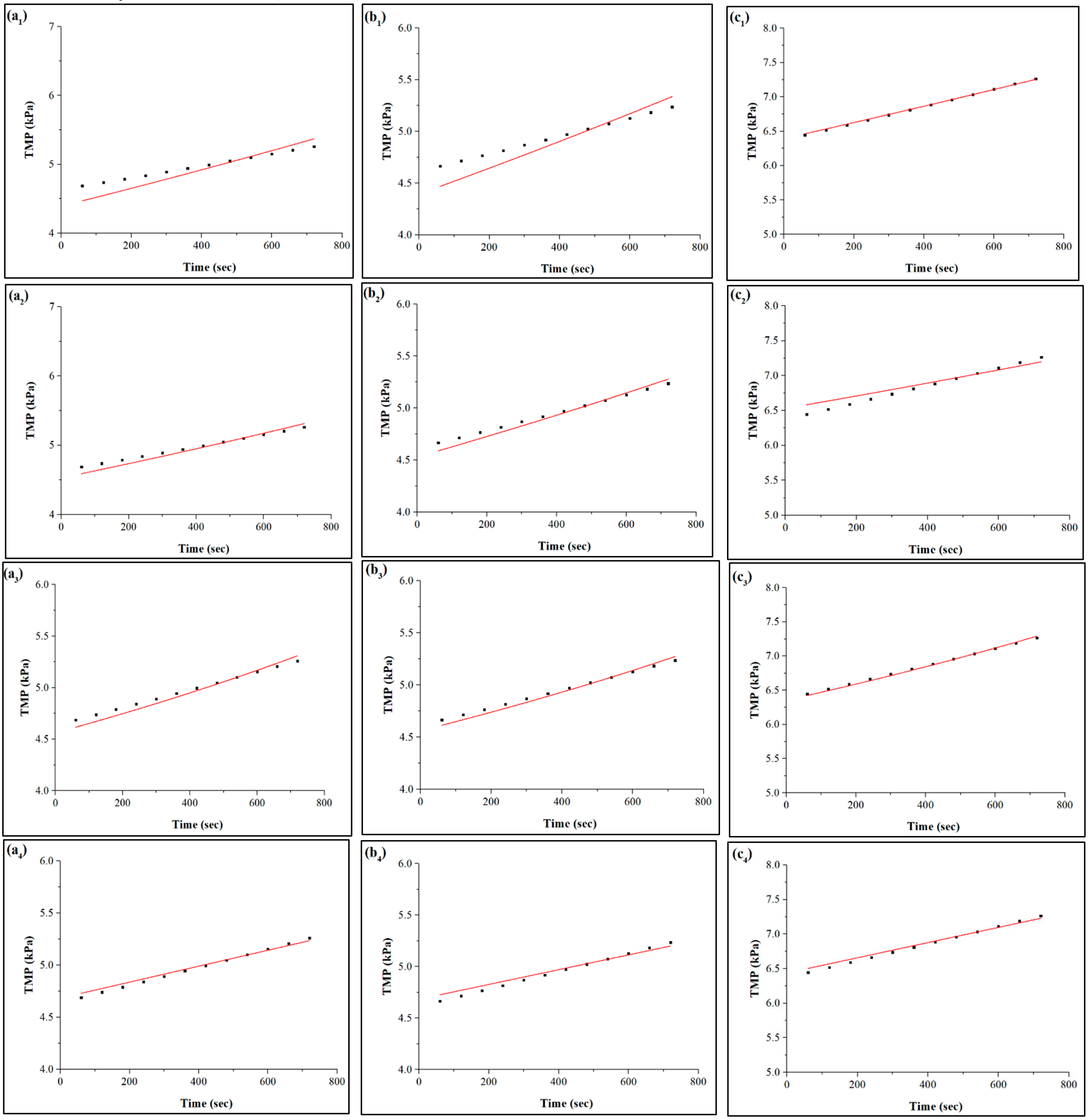

Fig. S15: Represents the fitted fouling curve for the fouling solution at 120lmh for cycle 3 filtration; a: S1 solution; b: S2 solution; c: S3 solution (subscript 1, 2,3 & 4 represents complete, intermediate, standard and cake layer blocking)

4. Fitting curves for modified fouling model for the 160lmh

4.1. 1hr Filtration

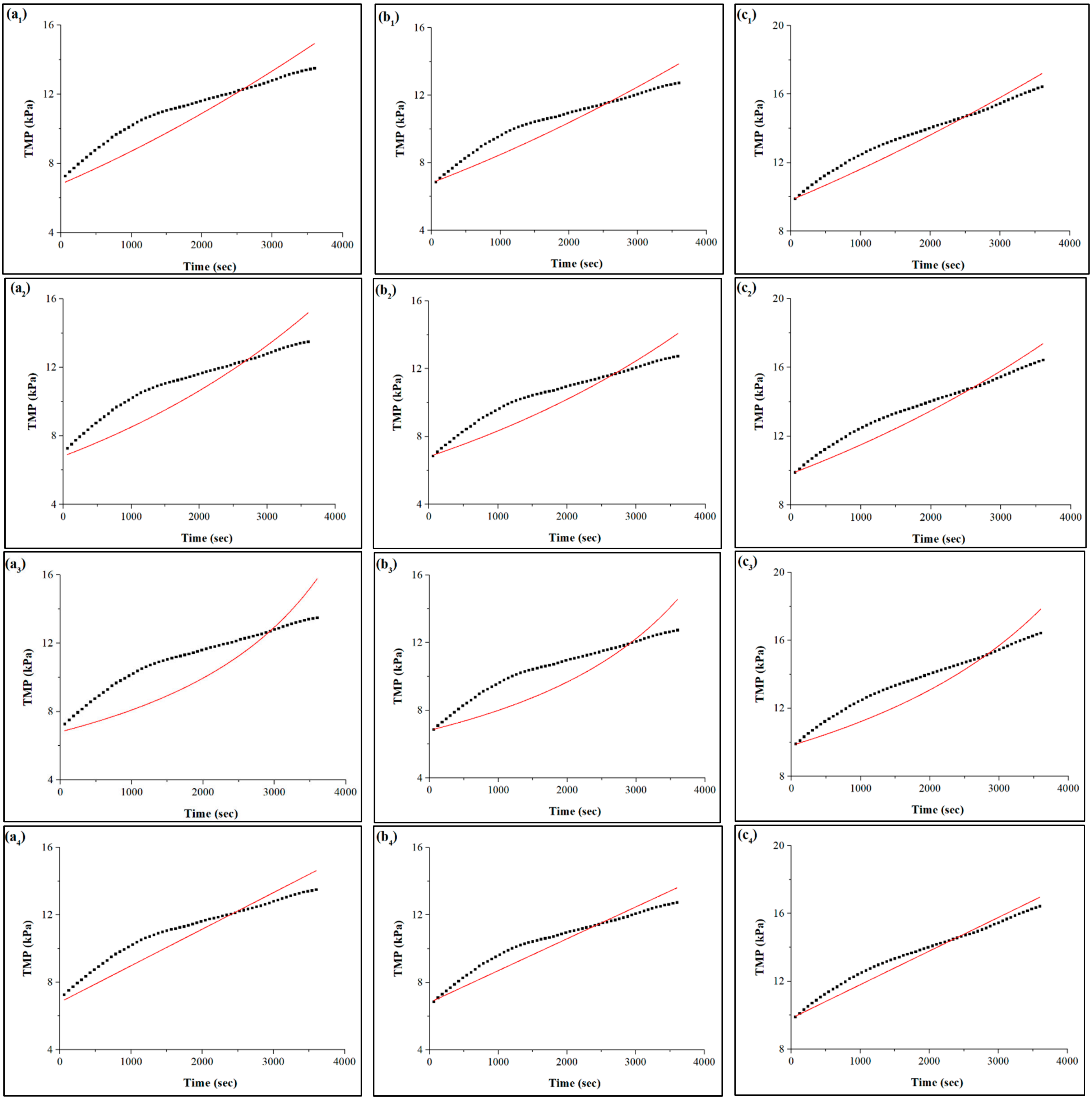

Fig. S16: Represents the fitted fouling curve for the fouling solution at 160lmh for 1hr filtration; a: S1 solution; b: S2 solution; c: S3 solution (subscript 1, 2,3 & 4 represents complete, intermediate, standard and cake layer blocking)

4.2.Cycle 1 filtration (160 lmh)

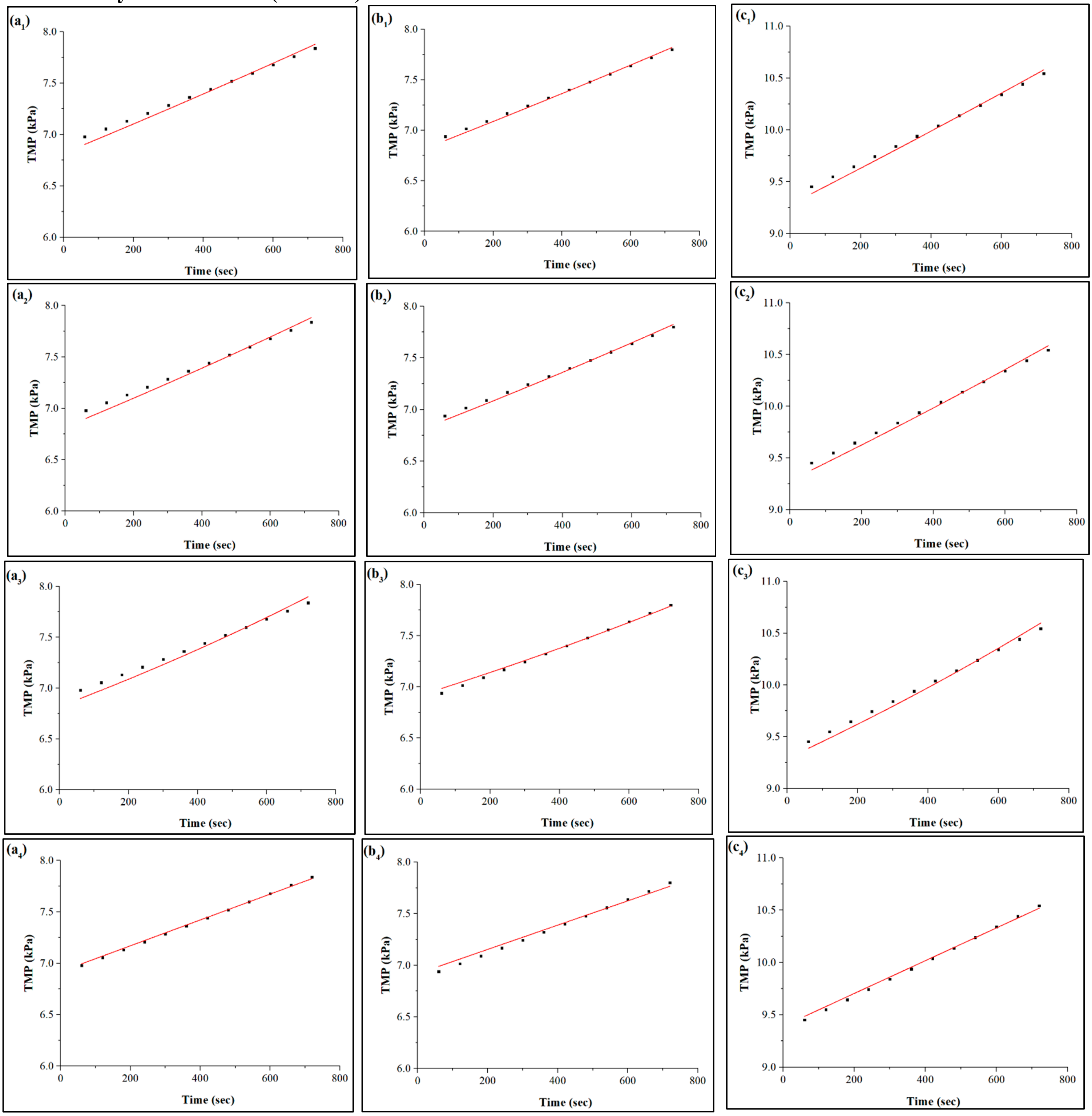

Fig. S17: Represents the fitted fouling curve for the fouling solution at 160lmh for Cycle 1 filtration; a: S1 solution; b: S2 solution; c: S3 solution (subscript 1, 2,3 & 4 represents complete, intermediate, standard and cake layer blocking)

4.3.Cycle 2 filtration (160 lmh)

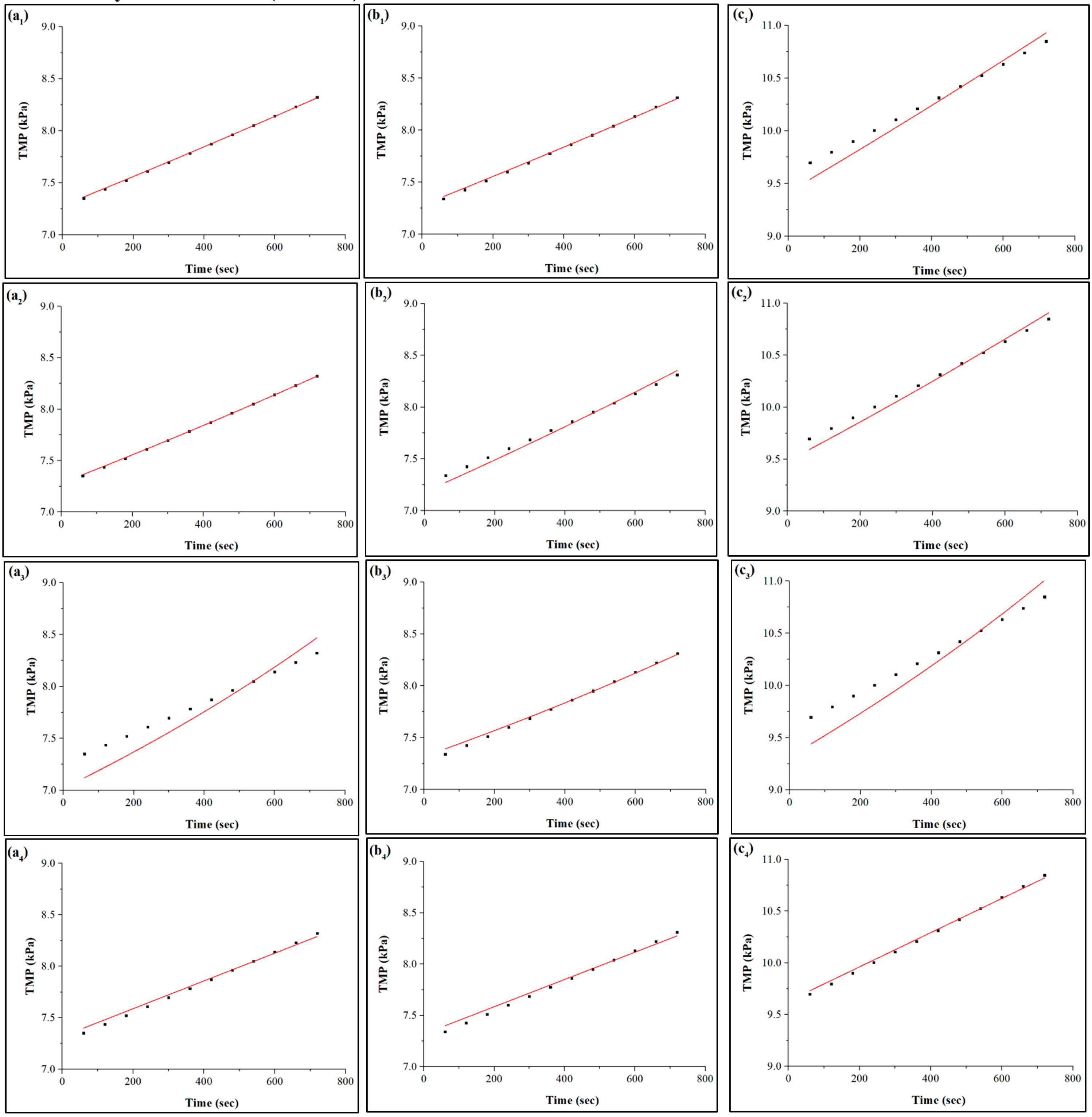

Fig. S18: Represents the fitted fouling curve for the fouling solution at 160lmh for Cycle 2 filtration; a: S1 solution; b: S2 solution; c: S3 solution (subscript 1, 2,3 & 4 represents complete, intermediate, standard and cake layer blocking)

4.4.Cycle 3 filtration (160 lmh)

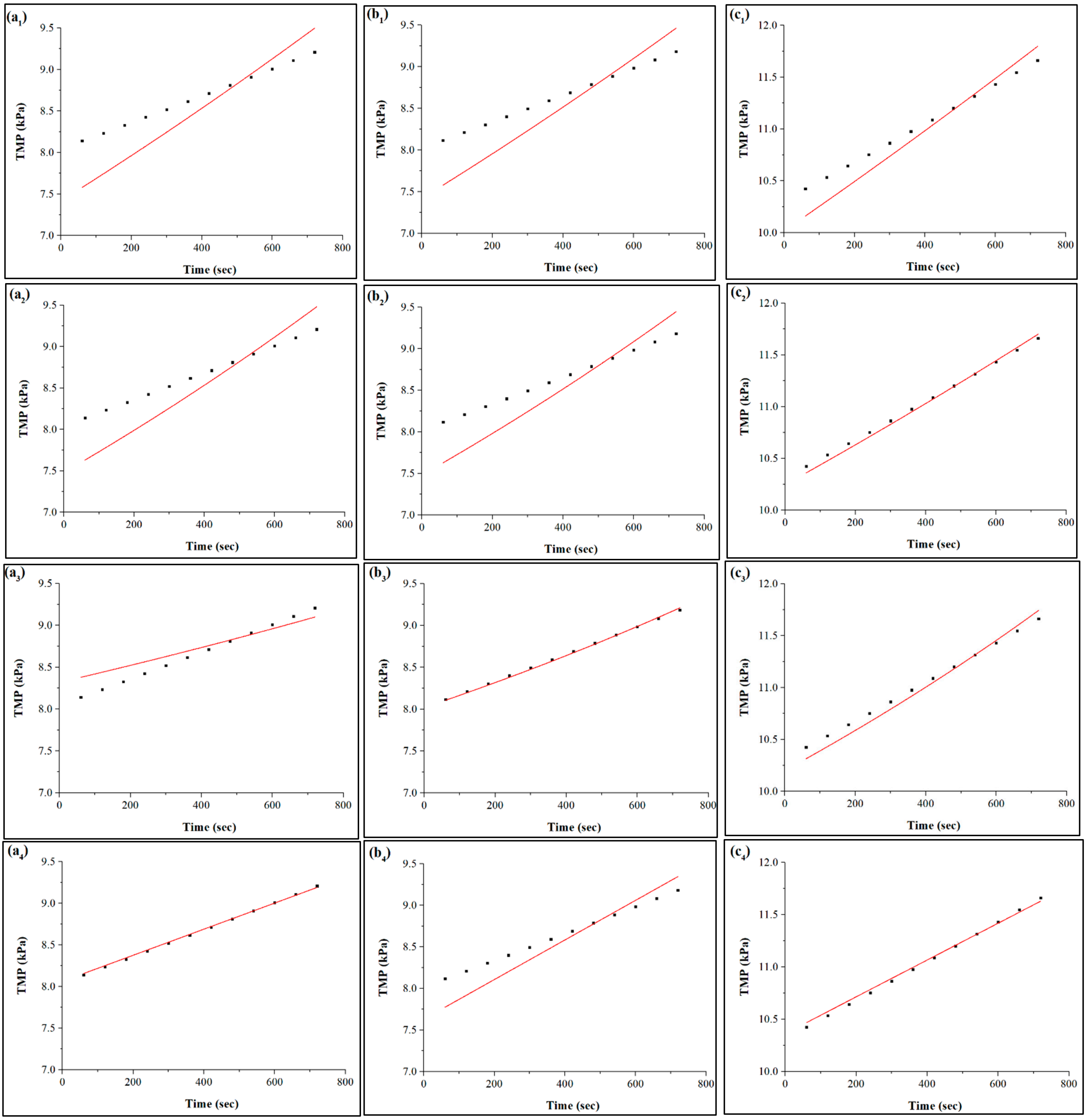

Fig. S19: Represents the fitted fouling curve for the fouling solution at 160lmh for Cycle 3 filtration; a: S1 solution; b: S2 solution; c: S3 solution (subscript 1, 2,3 & 4 represents complete, intermediate, standard and cake layer blocking)

5. Fitting curves for modified fouling model for the 120lmh

5.1.1hr Filtration

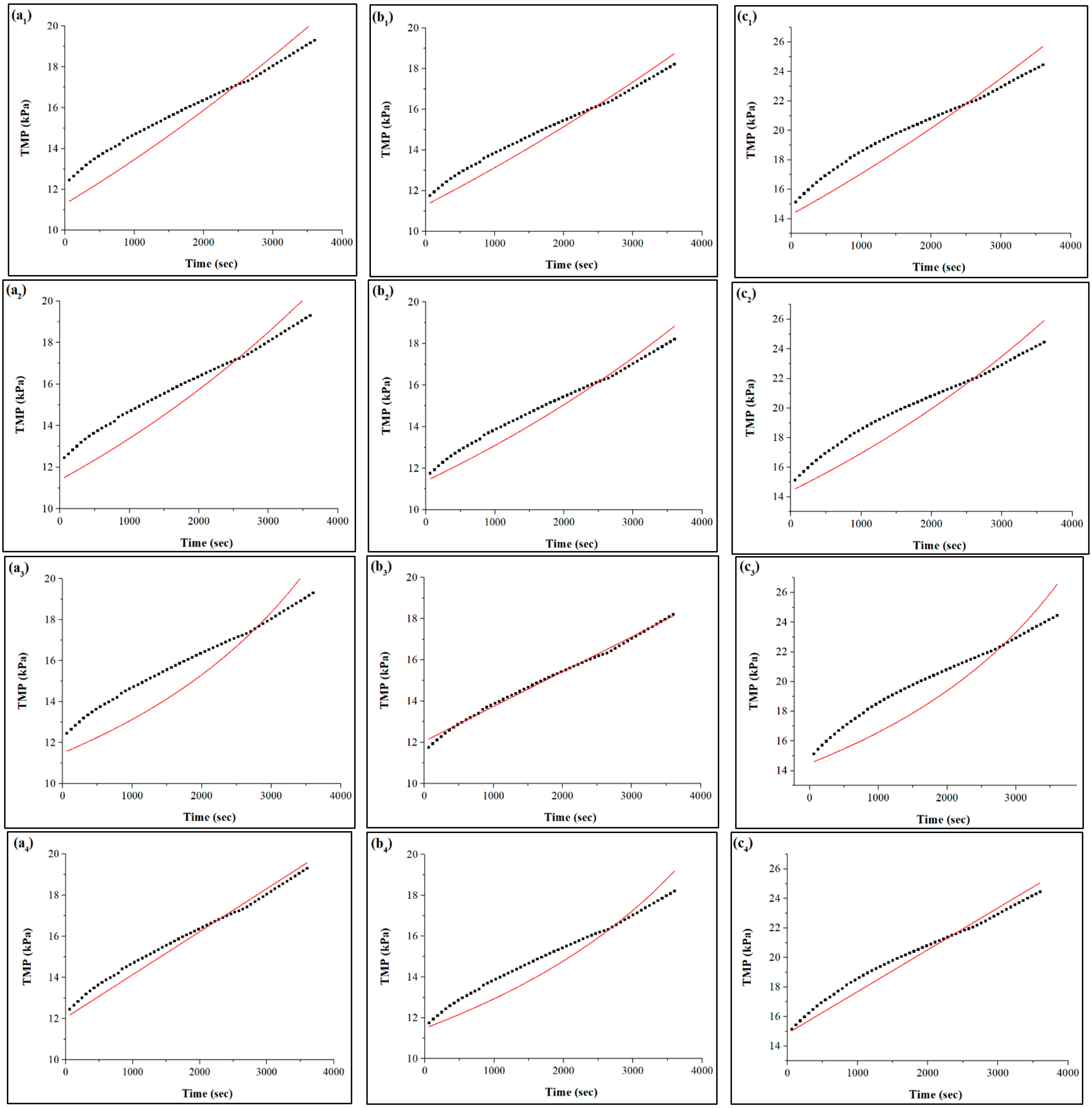

Fig. S20: Represents the fitted fouling curve for the fouling solution at 200lmh for 1hr filtration; a: S1 solution; b: S2 solution; c: S3 solution (subscript 1, 2,3 & 4 represents complete, intermediate, standard and cake layer blocking)

5.2.Cycle 1 filtration (160 lmh)

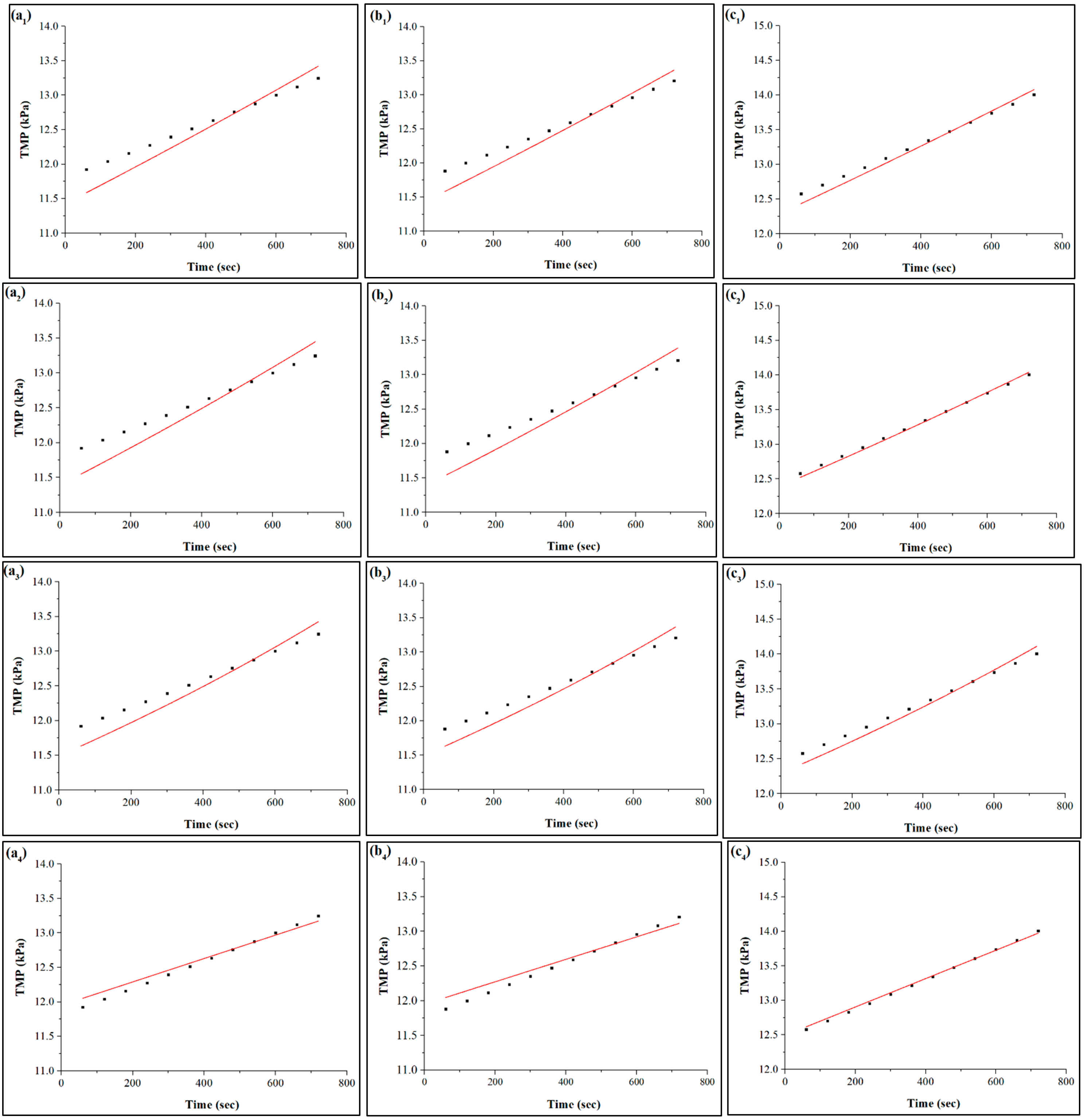

Fig. S21: Represents the fitted fouling curve for the fouling solution at 200lmh for Cycle 1 filtration; a: S1 solution; b: S2 solution; c: S3 solution (subscript 1, 2,3 & 4 represents complete, intermediate, standard and cake layer blocking)

5.3.Cycle 2 filtration (160 lmh)

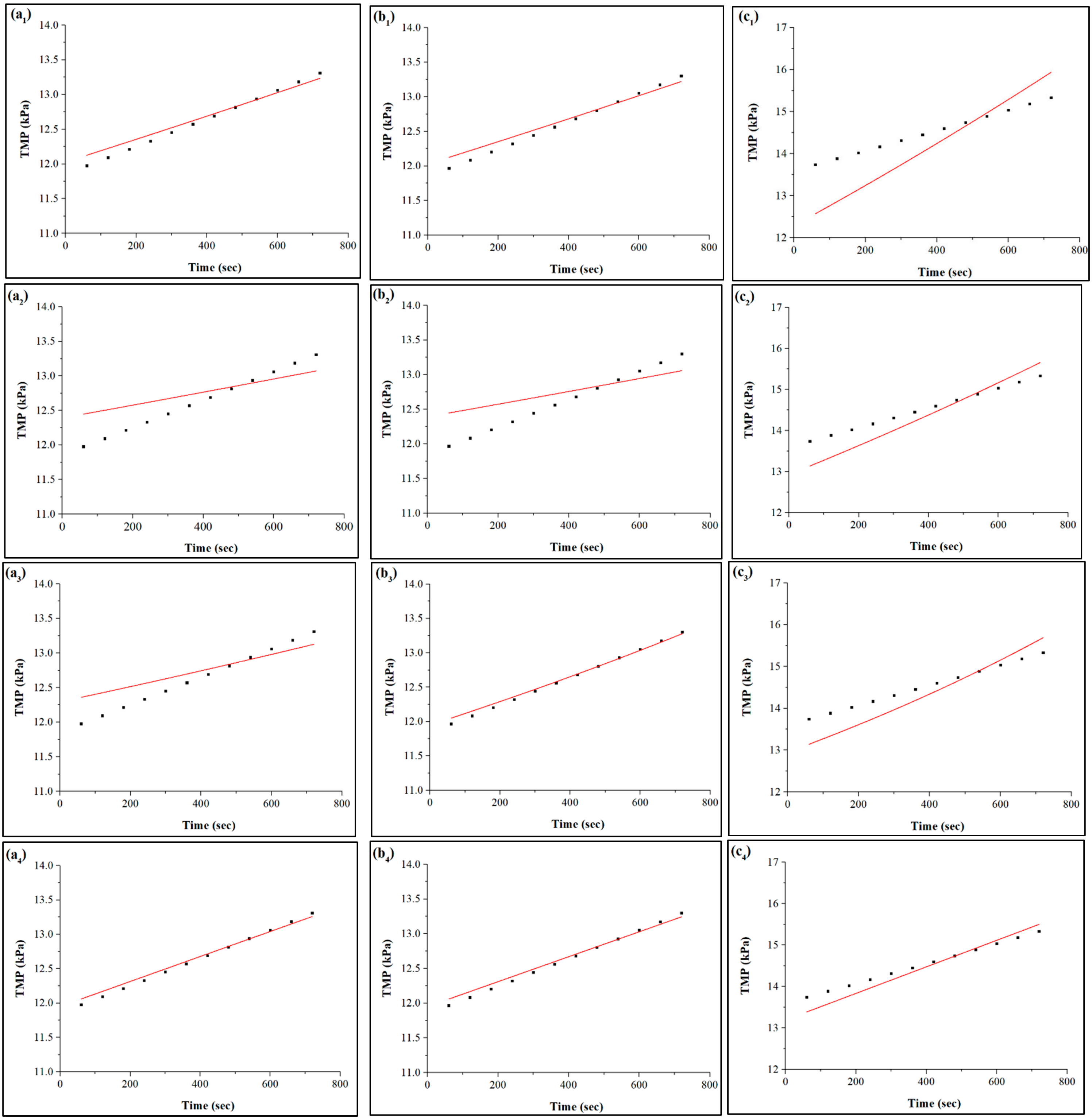

Fig. S22: Represents the fitted fouling curve for the fouling solution at 200lmh for Cycle 2 filtration; a: S1 solution; b: S2 solution; c: S3 solution (subscript 1, 2,3 & 4 represents complete, intermediate, standard and cake layer blocking)

5.4. Cycle 3 filtration (160 lmh)

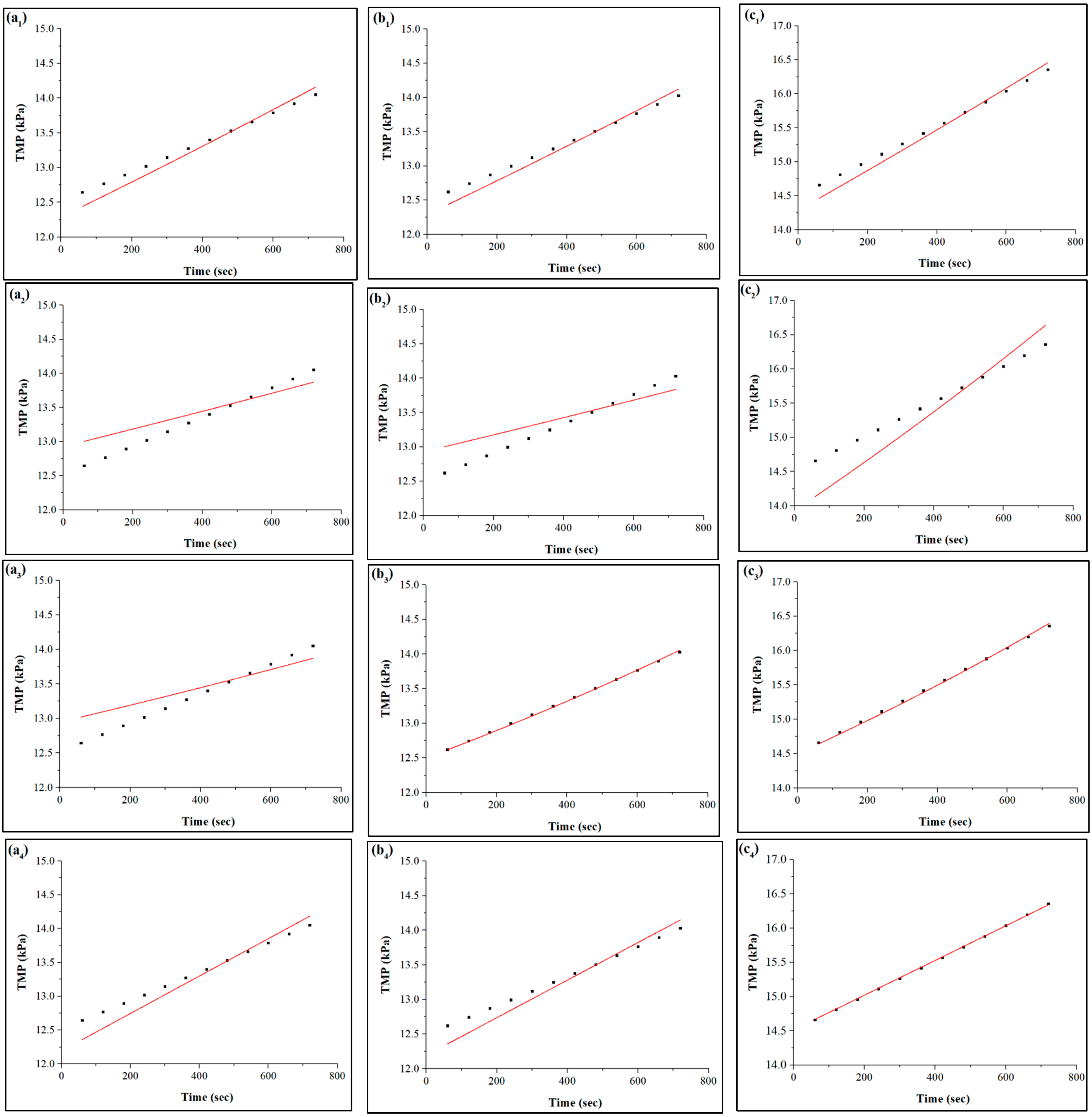

Fig. S23: Represents the fitted fouling curve for the fouling solution at 200lmh for Cycle 3 filtration; a: S1 solution; b: S2 solution; c: S3 solution (subscript 1, 2,3 & 4 represents complete, intermediate, standard and cake layer blocking)

Table S3: Summary of the foulants associated with membrane processes used for surface water treatment.

Membranes	Operational Parameters	Foulants		Results	Reference
		Organic	Inorganic		
PVDF	pH: 7: Temp: 23°	HA	Ca^{2+}, Mg^{2+}	• There is a decrease in the flux of 77% and 33% for Ca^{2+} and 53% and 72% for Mg^{2+} at ionic strengths of 1 mM and 50 mM, respectively	[1]
CA, PES	pH: 6.8 ± 0.2;; Temp: 25°C;	HA	Ca^{2+}, Mg^{2+}	• The flux decline rate is higher for PES (0.39 for Ca; 0.44 for Mg) than for CA (0.63 for Ca; 0.80 for Mg) due to the strong hydrophobic interactions of the complexes formed from the HA–HA-divalent cation reaction because PES is more hydrophobic (contact angle: 91.9^{o}).	[2]
PES (20 kDa)	P: 105 ± 2.07 kPa	DOC	Ca^{2+}; Mg^{2+}	• Intermolecular bridging of Ca^{2+}, Mg^{2+} ion between NOM leads to severe fouling due to strong adhesion forces, resulting in the severe decline in membrane flux..	[3]
PVDF (0.22 µm)	pH: 6.5–7.5; Temp: 35–40°C	HA	Fe^{3+}	• Fe is not effective as a coagulant for the removal of viruses from surface water even at high dosages of 13 $mg.L^{-1}$.	[4]
PVDF (0.1 µm),	pH: 12; Temp: 20°C	DOC	Al^{3+} , Mn^{2+}; Fe^{3+}; Ca^{2+}	• Evaluation of foulant removal by particle size is carried out, showing that Al and Fe have larger particle sizes than Mn, so they were efficiently removed.	[5]
PA (0.6 kDa)	pH: 6.5–7; Temp: 22±3°C	HA, FA	Ca^{2+}	• PA membranes are used to analyze DOM fouling with and without Ca^{2+}. Membrane fouling is not reported only according to hydrophilicity/hydrophobicity, but the surface chemistry is also considered to determine the strength of foulant–membrane interactions.	[6]
PES (100 kDa)	pH: 7.5 ±0.1; Temp: 21± 2°C	HA; PAC	Ca^{2+}	• In the presence of calcium, the TFI and HIFI are at least 2 times higher in PAC+HA than in PAC-treated HA.	[7]
PVDF	pH: 7; Temp: 20°C	DOC	Na^{+1} ; Mg^{2+}	• HA fouling with Na^{2+} is dominated by electrostatic forces, leading to the formation of a thick layer of HA on the surface with an increase in concentration. Mg^{2+} exhibits different behavior, with an increase in the concentration to a critical value causing the aggregation of HA due to electrostatic forces	[8]
PES, CA, PVDF; (100 kDa)	Temp: 21 ± 2°C; pH: 7.5 ± 0.1	HA; SA BSA;PAC	Ca^{2+}	• The mass of PAC increases from 0.65 and 0.41 $g.m^{-2}$ to 3.33 and 2.65 $g.m^{-2}$ when Ca^{2+} combines with HA and SA, respectively, due to the interaction between the carboxylate moieties of HA and SA with Ca^{2+}, further strengthening adsorption.	[9]
PVC (50 kDa)	pH: 6.8 ± 0.1; Temp: 23-25°C	HA	Ca^{2+}	• HA fouling is dependent on Ca^{2+} interactions. As the Ca^{2+} concentration increases, membrane fouling increases due to chelation and a decrease in the interaction energy with HA.	[10]
PVDF	pH:8 Temp: 23-25°C	HA	Ca^{2+}, Al^{3+}, Mn^{2+}, Fe^{3+}	• Applied the participation equation to elucidate the dual behavior of Al^{3+} with HA and Ca^{2+}, while bridging and agglomeration with other foulants.	This study

References

1. Miao, R., et al., *A comparison of the roles of Ca2+ and Mg2+ on membrane fouling with humic acid: Are there any differences or similarities?* Journal of Membrane Science, 2018. **545**: p. 81-87.
2. Wang, L.-F., et al., *Probing the roles of Ca2+ and Mg2+ in humic acids-induced ultrafiltration membrane fouling using an integrated approach.* Water Research, 2015. **81**: p. 325-332.
3. Ahn, W.-Y., A.G. Kalinichev, and M.M. Clark, *Effects of background cations on the fouling of polyethersulfone membranes by natural organic matter: Experimental and molecular modeling study.* Journal of Membrane Science, 2008. **309**(1): p. 128-140.
4. Tanneru, C.T. and S. Chellam, *Mechanisms of virus control during iron electrocoagulation – Microfiltration of surface water.* Water Research, 2012. **46**(7): p. 2111-2120.
5. Yamamura, H., K. Kimura, and Y. Watanabe, *Mechanism Involved in the Evolution of Physically Irreversible Fouling in Microfiltration and Ultrafiltration Membranes Used for Drinking Water Treatment.* Environmental Science & Technology, 2007. **41**(19): p. 6789-6794.
6. Mustafa, G., et al., *New insights into the fouling mechanism of dissolved organic matter applying nanofiltration membranes with a variety of surface chemistries.* Water Research, 2016. **93**: p. 195-204.
7. Shao, S., et al., *Deposition of powdered activated carbon (PAC) on ultrafiltration (UF) membrane surface: influencing factors and mechanisms.* Journal of Membrane Science, 2017. **530**: p. 104-111.
8. Miao, R., et al., *Evaluating the effects of sodium and magnesium on the interaction processes of humic acid and ultrafiltration membrane surfaces.* Journal of Membrane Science, 2017. **526**: p. 131-137.
9. Shao, S., et al., *Combined influence by humic acid (HA) and powdered activated carbon (PAC) particles on ultrafiltration membrane fouling.* Journal of Membrane Science, 2016. **500**: p. 99-105
10. Lin, T., Z. Lu, and W. Chen, *Interaction mechanisms of humic acid combined with calcium ions on membrane fouling at different conditions in an ultrafiltration system.* Desalination, 2015. **357**: p. 26-35.
